# Synthetic Mechanism of a Fe(II) *N*‑Heterocyclic Carbene Bidentate Complex Revealed by Electronic Structure Methods

**DOI:** 10.1021/acs.inorgchem.6c02095

**Published:** 2026-06-22

**Authors:** Abdelazim M. A. Abdelgawwad, Ulises Carrillo, Philippe C. Gros, Cristina Cebrián, Antonio Francés-Monerris

**Affiliations:** † Institut de Ciència Molecular, 16781 Universitat de València, P.O. Box 22085, València 46071, Spain; ‡ Université de Caen Normandie, CERMN UFR 4258, 14000 Caen, France; § Université de Grenoble Alpes, CNRS, DCM, F-38000 Grenoble, France; ∥ Université de Strasbourg, CNRS, ICS, F-67000 Strasbourg, France

## Abstract

Octahedral Fe­(II) complexes with bidentate *N*-heterocyclic (NHC) ligands are solid candidates for photoactive materials based on first-row transition metals. Despite the remarkable advances in ligand design and excited-state control, the theoretical basis for describing the complexation mechanism from a molecular and electronic point of view is lacking. This work reveals the molecular motions that drive the formation of bidentate [Fe­(C^N)_3_]^2+^ complexes and how they couple with the electronic structure. Quantum chemistry methods are used to describe the chemical reactions that lead to the [Fe­(pyIm)_3_]^2+^ (pyIm = pyridine-imidazol-2-ylidene) complex as a model case. The molecular model employed is based on the canonical synthesis using FeCl_2_ in an organic solvent and a strong Brønsted base to generate the pyIm ligand *in situ*. The energy profiles indicate that almost all reactivity takes place in the quintet state, whereas the singlet ground state is only populated in the last coordination step. Both d-activated dissociative interchange (I_d_) and purely dissociative (D) mechanisms compete, although the former is expected to be slightly more favorable. A global description of the coordination mechanism, consistent with the available experimental data, is provided through an analysis of the kinetic competition between the pathways and the thermodynamic stability of the intermediates.

## Graphical abstract

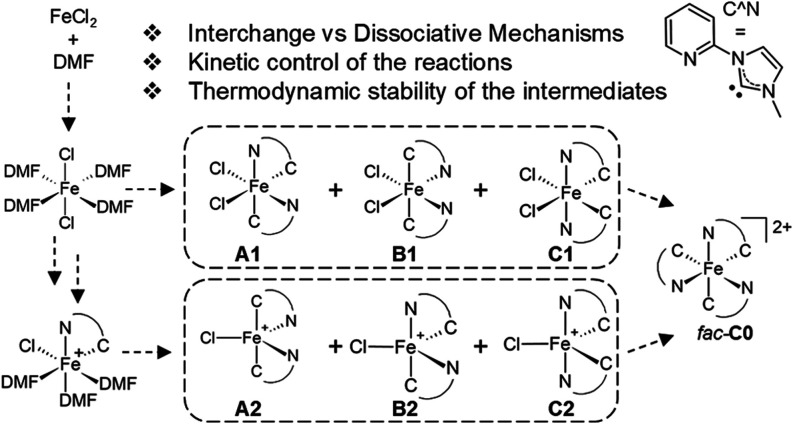

## Introduction

Coordination compounds based on transition metals are crucial across many branches of chemistry and technology due to their convenient photophysical, magnetic, and electrochemical properties.^1–6^ These compounds are at the basis of paramount industrial processes and applications such as light-emitting devices,^1,7–9^ (photo)­catalysis,^10–15^ photodynamic therapy,^16,17^ and radiomedicine and bioimaging,^18,19^ representing a very active field of research in both academy and industry.

In the last years, there has been a great interest in the design of cheap and green photoactive materials based on first-row transition metals able to replace traditional chromophores based on rare and precious elements such as ruthenium or iridium.^11,20–32^ In this context, iron constitutes an appealing candidate as abundant, cheap, and relatively nontoxic metal.^33,34^ Typical polypyridyl octahedral Fe­(II) complexes populate metal-to-ligand charge-transfer (MLCT) electronic states upon light absorption, even though their relatively small splitting of the d metal orbitals give rise to the presence of low-lying metal-centered (MC) states, favoring the ultrafast deactivation of the photoactive MLCT states.^34–39^ In this regard, the use of strong σ-donating *N*-heterocyclic carbene (NHC) ligands represents a crucial milestone in the search for photoactive ferrous compounds. Indeed, these ligands have proven instrumental in sufficiently destabilizing the MC states, extending MLCT lifetimes from tens to hundreds of picoseconds,^4,40–42^ well beyond the ultrashort subpicosecond scale of the archetype compound [Fe­(bpy)_3_]^2+^.^33,43–46^ Even lifetimes in the nanosecond regime have been attained, though with a cyclometalated complex.^47^ These outstanding achievements have spurred the application of Fe­(II)-based chromophores in different fields, resulting particularly useful in solar energy conversion^43,45,48^ and photocatalysis.^49^

Geometrical aspects of ligand coordination exert a direct modulation of the interaction with the metal. For instance, bidentate azine-NHC ligands offer a more adequate coordination with Fe­(II) than their tridentate analogs, as reflected by their longer-lived MLCT states.^50^ Interestingly, even the specific arrangement of bidentate azine-NHC ligands, i.e., facial (*fac*) or meridional (*mer*) configurations, results in distinct excited stated properties, with facial isomers exhibiting slower MLCT relation kinetics.^4,50,51^

Apart from the electronic features of the coordinating units, steric ligand demand and secondary interactions are also key modulating elements in the intricate photophysical landscape of NHC-based Fe­(II) complexes. For instance, Kupfer, Dietzek-Ivanšić, Sorsche and colleagues recently reported that peripheral ligand protonation can alter the relative stabilization of ^3^MLCT vs ^3^MC states.^52^ In the case of the work of McCusker, Herbert and co-workers, the increased rigidity afforded by interligand π-stacking in combination with the strong σ-donation of the ligands trapped the complex in the elusive ^3^MC state for several nanoseconds prior to its ground state relaxation.^53^

Starting chemicals to synthesize the octahedral NHC-based complexes such as [Fe­(C^N)_3_]^2+^ or [Fe­(C^N^C)_2_]^2+^ vary across the available literature and research groups.^54^ The most common approach consists in the treatment of the imidazolium ligand precursor with commercially available iron salts such as FeCl_2_,^50,55–59^ FeBr_2_,^53,60^ or Fe­(OTf)_2_ ^47^ followed by the addition of a strong base, usually *t*-BuOK or KHMDS (HMDS = hexamethyldisilazide),^50,53,55–58,60^ to generate the NHC ligand. Scheme 1a shows the standard procedure reported by Gros, Cebrián, and co-workers^4,50,57^ in which FeCl_2_ is used as iron source and the NHC molecule **L0** is generated *in situ* by the reaction of **HL0** with *t*-BuOK (Scheme 1b). As a result of the strong donor character of NHC, + II oxidation state becomes less stable as the number of carbenic units in the coordination sphere increases. This can be illustrated by the hexacarbene complex [Fe­(btz)_3_]^2+^ (btz = 3,3′-dimethyl-1,1′-bis­(*p*-tolyl)-4,4′-bis­(1,2,3-triazol-5-ylidene))^40^ described by Wärnmark and co-workers, which was prepared upon reduction of the corresponding ferric analogue [Fe­(btz)_3_]^3+^.^27^ Another consequence of the carbenic donor strength is the high *trans*-effect of this unit, that favors the preparation of homoleptic complexes. However, heteroleptic complexes can be synthesized as well from polyimine intermediates^55^ or the use of Fe­(HMDS)_2_ as both iron and base source.^52,61^

**Scheme 1:**
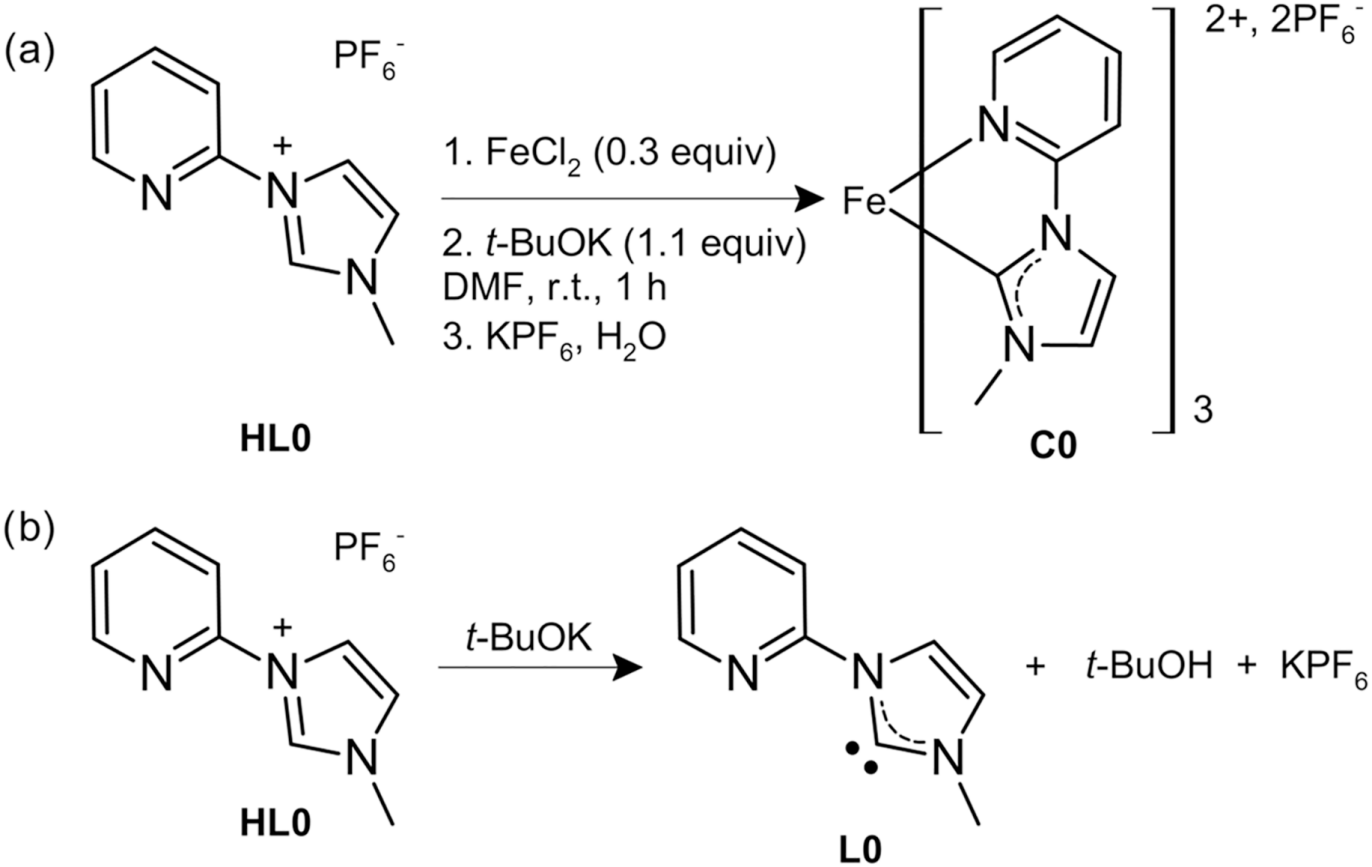
Synthesis of the [Fe­(pyIm)_3_]^2+^ Complex **C0** Studied in This Work (Panel a) and Generation of the C^N Ligand **L0** in the Synthesis Medium (Panel b)^50,57^

Contrary to tridentate ligands, unsymmetrical bidentate ligands give rise to geometric isomers. This can be illustrated with the synthesis of complex **C0**, which is obtained as a mixture of 1:14 *fac*:*mer* isomers that proved difficult to resolve.^57^ Several ligand modifications with varying azine and NHC units were subsequently explored, resulting in isomer ratio variations until complete stereoselection in some cases.^4,56^ Nevertheless, despite its paramount relevance, the complexation mechanism of azine-NHC units to the Fe center still lacks a theoretical basis.

Several mechanisms can be invoked for ligand exchange reactions of solvated metal ions (Scheme 2).^62,63^ In a pure dissociative (D) mechanism, the ligand to be replaced leaves the coordination sphere to form an intermediate with a decreased coordination number that later forms the bond with the incoming ligand. In a pure associative (A) mechanism, the process is reversed, and the rate-limiting step is the formation of an intermediate with an increased coordination number that further evolves breaking the bond of the leaving ligand.^62^ There is, however, a continuum of intermediate mechanisms in between the two D and A extreme cases, in which both the bond formation with the entering ligand and the bond breaking with the leaving ligand make varying contributions to the transition state energetics, known as interchange (I) mechanisms (Scheme 2).^62^ Kinetic measurements at various pressures of the ligand exchange reaction of [Fe­(DMF)_6_]^2+^ revealed that the activation volume Δ*V* ^‡^ is positive,^64^ indicating that the process proceeds by an interchange mechanism dominated by dissociation, namely a d-activated dissociative interchange (I_d_) mechanism, in which the nature of the substituting ligand barely influences the substitution kinetics.^62^

**Scheme 2:**
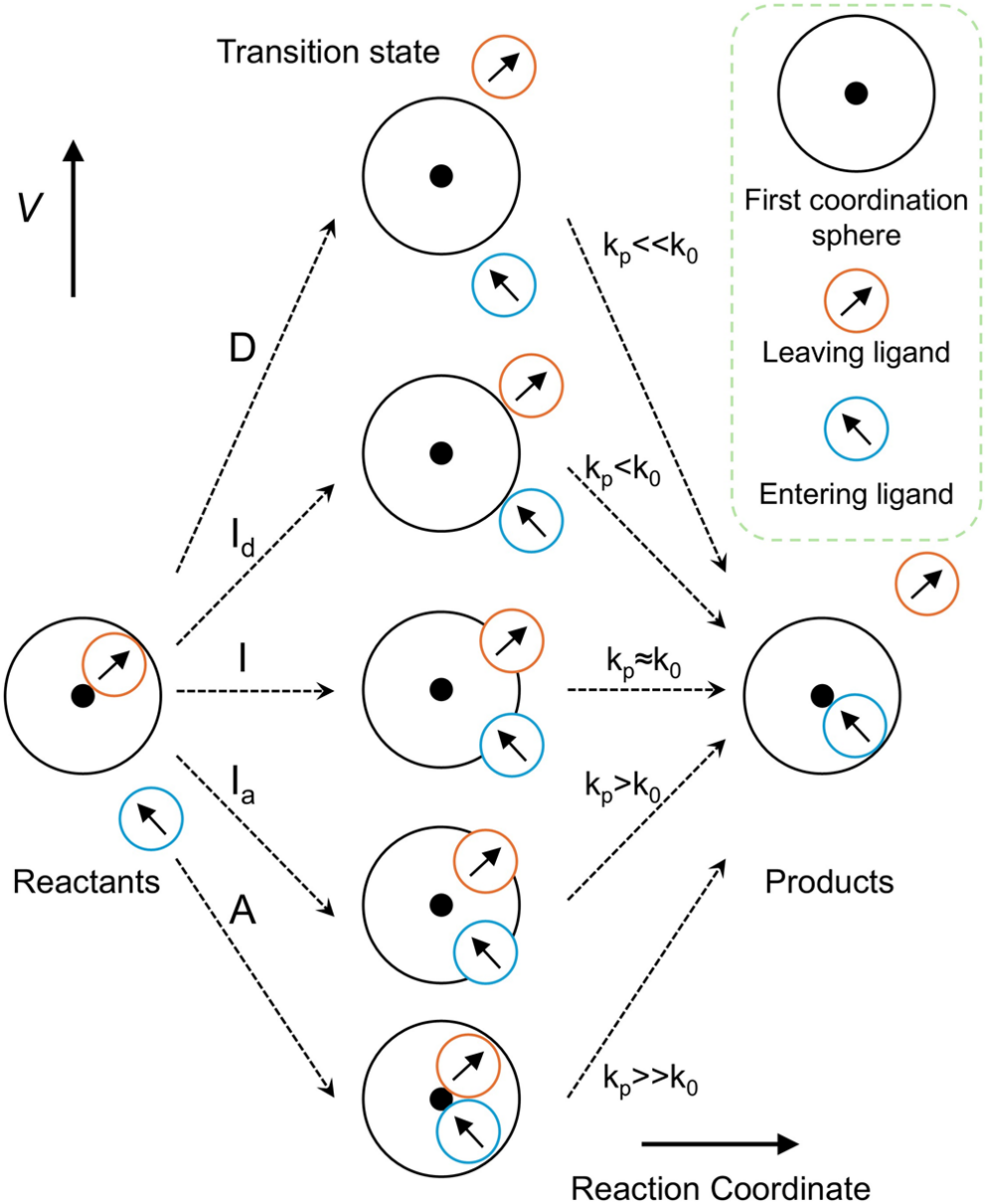
Schematic Representation of the Dissociative (D), Dissociative-Activated Interchange (I_d_), Interchange (I), Associative-Activated Interchange (I_a_), and Associative (A) Mechanisms for Ligand Substitution Reactions[Fn s2fn1]

a
*V* stands for molar volume while the ligand exchange kinetic constants at ambient and high pressure are represented by *k*_0_ and *k*_p_, respectively.^62^ Figure reprinted and edited with permission from ref 62. Copyright 1995 Elsevier.

By using quantum chemistry methods, this work reveals, for the first time, the complexation of the C^N ligand **L0** (Scheme 1b) to produce the complex [Fe­(pyIm)_3_]^2+^ **C0** ^50,57^ as a model case of [Fe­(C^N)_3_]^2+^ synthetic mechanism (Scheme 1). All precomplex intermediates are formed in a high spin (quintet), while the singlet ground state is generated only in the last step of the coordination process (Fe–N formation). This synthesis serves as an ideal prototype to rationalize due to the canonicity of the procedure, which uses FeCl_2_ as iron source, the structural simplicity of **L0**, and the solvent popularity (DMF). The XYZ coordinates of the most important reaction paths can be found in the Supporting Information.

## Results and Discussion

### Fe­(II) Solvocomplex in DMF

FeCl_2_ is an ionic^65,66^ and highly hygroscopic solid soluble in water and in organic coordinating solvents.^67–69^ However, the characterization of the Fe­(II) molecular solvocomplexes is much less documented than its Fe­(III) analogue, which can be partially attributed to their higher lability and tendency to oxidize in air.^69^ Available literature on FeCl_3_ indicates that it produces a wide variety of octahedral complexes such as [FeL_4_Cl_2_]^+^ (L= H_2_O, DMF, DMSO) and *mer*-[FeL_3_Cl_3_] (L= pyridine, pyrazole, *N*-methylimidazole) in solution, where chlorine atoms remain in the coordination sphere.^70^ In this regard, the capacity of organic solvent molecules to coordinate anhydrous FeCl_2_ is expected to be similar to that of FeCl_3_ as illustrated by solvocomplexes like *trans*-[FeL_4_Cl_2_] in aqueous^71^ or pyridine^72^ solutions. For instance, Mandon and collaborators^73^ mixed bis­[2-(2,3-dihydroxyphenyl)-6-pyridylmethyl]­(2-pyridylmethyl)­amine (BCATTPA) with FeCl_2_ and FeCl_3_ in ACN or THF, resulting in the octahedral [Fe­(BCATTPA)­Cl_2_]*^n^* (*n* = 0 and +1 for Fe^2+^ and Fe^3+^, respectively) complexes. The Fe­(II) synthesis is described in Scheme 3 and shows the *cis* disposition of the Cl^–^ ligands.

**Scheme 3:**
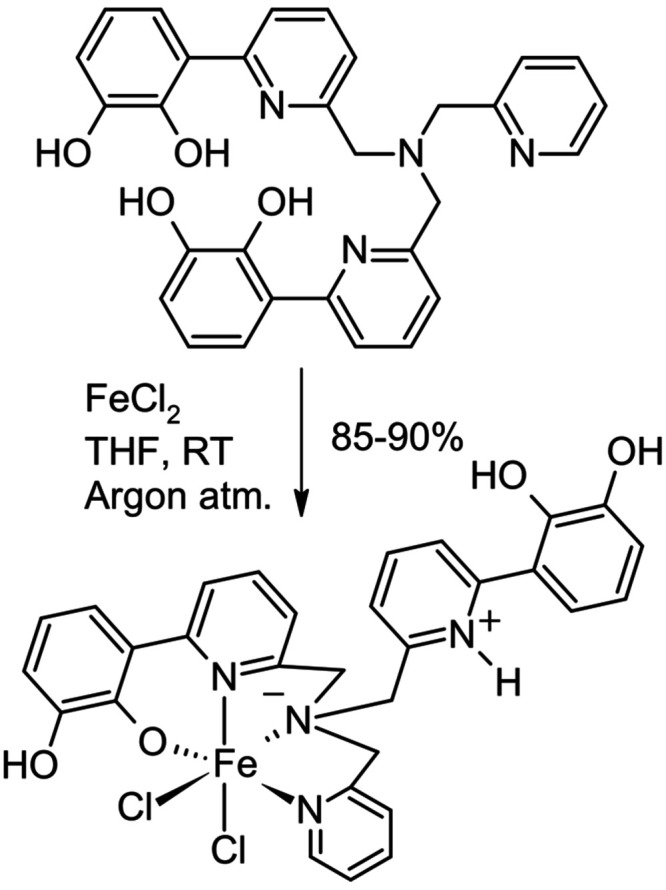
Synthesis and Structure of the Fe­(II) Complex [Fe­(BCATTPA)­Cl_2_] Reported by Machkour et al.^73^

On the basis of the aforementioned evidence, the dominant molecular species formed on solubilizing FeCl_2_ in DMF are likely the solvocomplexes *cis*- or *trans*-[FeCl_2_(DMF)_4_] (reaction 1), in which the two chloride atoms remain coordinated to iron:

1 $${\mathrm{FeCl}}_{2}+4\mathrm{DMF}\to \mathrm{trans}‐\left[{\mathrm{FeCl}}_{2}{\left(\mathrm{DMF}\right)}_{4}\right]+\mathrm{cis}‐\left[{\mathrm{FeCl}}_{2}{\left(\mathrm{DMF}\right)}_{4}\right]$$

Both DFT and DLPNO–CCSD­(T) methods used in this work predict the first quintet (Q_1_) as the electronic ground state of FeCl_2_ and *cis*/*trans*-[FeCl_2_(DMF)_4_], as shown in Table S1. Thus, the complexes are considered to be high-spin species as a result of the small ligand field.^74^ Among the two possibilities, the *trans* isomer is more stable than the *cis* disposition at the B3LYP/6–31G* level of theory (*G*(*cis*) – *G*(*trans*) = 1.49 kcal/mol). Therefore, the former is considered as the dominant product of reaction 1.

### Absence of Ligand Precoordination at Room Temperature

Reaction 1 takes place *before* the addition of the strong base (*t*-BuOK) and the subsequent formation of **L0** (Scheme 1), leading to the coexistence of the [FeCl_2_(DMF)_4_] and **HL0** species in the reaction media. The possibility of a precoordination of the pyridine moiety before the carbene generation was ruled out by Magra on the basis of UV–vis spectroscopy measurements over time.^75^ No spectral changes in the FeCl_2_ solvocomplex in the presence of **HL0** were detected up to 60 min after mixture of the reactants at room temperature (Figure S1a), whereas the buildup at ∼370 nm and the depletion at ∼310 nm at 110 °C suggest a clean chemical reaction (Figure S1b), likely the transformation of [FeCl_2_(DMF)_4_] to other complexes of unknown structure due to coordination of the pyridine moiety of **HL0**. The mixture of FeCl_2_ with standalone pyridine at room temperature induces similar spectral changes (Figure S1c), reinforcing the pyridine complexation of **HL0** only at high temperature (110 °C). Therefore, since no pyridine precoordination was found at room temperature,^75^ the ligand coordination to FeCl_2_ in this work is considered to take place only once **L0** is formed.

### d-Activated Dissociative Interchange Complexation Sequence

Subsequent complexation via ligand substitution is well-known to take place sequentially,^74^ in which the strong *trans*-effect of both pyridine and carbene units drives the substitution of the DMF ligands (reactions 2 and 3).^76^ As shall be discussed below, the Cl^–^ released in reaction 2 replaces a DMF in [FeCl­(C^N)_2_(DMF)]^+^ (reaction 4), in line with complexes of the type [Fe­(N^N)_2_X_2_][Bibr ref77] and the *cis*-[Fe­(C^N)_2_(CH_3_CN)_2_] intermediate reported by Kupfer, Dietzek-Ivanšić, Sorsche and colleagues.^52^ This finding proves that the steric interactions between the α-hydrogen atom in the pyridine ring and the N–CH_3_ group in the carbene moiety are high enough to preclude the formation of the *trans* isomer.

2 trans‐[FeCl2(DMF)4]+C^N→trans,cis‐[FeCl(C^N)(DMF)3]++DMF+Cl−

3 trans,cis‐[FeCl(C^N)(DMF)3]++C^N→[FeCl(C^N)2DMF]++2DMF

4 [FeCl(C^N)2DMF]++Cl−→cis,xxx‐[FeCl2(C^N)2]+DMF

5 cis,xxx‐[FeCl2(C^N)2]+C^N→fac‐[Fe(C^N)3]2++mer‐[Fe(C^N)3]2++2Cl−

The [Fe­(C^N)_3_]^2+^ complex **C0** ([Fe­(pyIm)_3_]^2+^) is formed in reaction 5. As for the relative orientation (xxx) of C^N, while irrelevant in *trans*,*cis*-[FeCl_2_(C^N)­(DMF)_2_], it plays an important role within the neutral intermediate formed in (4). In fact, the *fac* or *mer* isomerism of **C0** is determined upon introducing the last C^N ligand in reaction 5.

### Purely Dissociative Complexation Sequence

This work also explores the possibility of a D mechanism, in which bond breaking of the leaving ligand is complete prior bond formation of the entering ligand (Scheme 2). These processes compete with the I_d_ reactions 2–5 and are envisaged as follows:

6a $$\mathrm{trans}‐\left[{\mathrm{FeCl}}_{2}{\left(\mathrm{DMF}\right)}_{4}\right]\to \mathrm{trans}‐[\mathrm{FeCl}{\left(\mathrm{DMF}\right)}_{4}{]}^{+}+{\mathrm{Cl}}^{-}$$

6b $$\mathrm{trans}‐\left[{\mathrm{FeCl}}_{2}{\left(\mathrm{DMF}\right)}_{4}\right]\to \mathrm{trans}‐\left[{\mathrm{FeCl}}_{2}{\left(\mathrm{DMF}\right)}_{3}\right]+\mathrm{DMF}$$

7 trans‐[FeCl2(DMF)3]+C^N→[FeCl(C^N)(DMF)3]++Cl−

8a [FeCl(C^N)(DMF)3]+→[FeCl(C^N)(DMF)2]++DMF

8b [FeCl(C^N)(DMF)3]+→[Fe(C^N)(DMF)3]2++Cl−

9 [FeCl(C^N)(DMF)2]++C^N→[FeCl(C^N)2(DMF)]++DMF

Reaction 4, which is common to both mechanisms, takes place after reaction 11 to yield *cis*,*xxx*-[FeCl_2_(C^N)_2_].

10 cis,xxx‐[FeCl2(C^N)2]→[FeCl(C^N)2]++Cl−

11 [FeCl(C^N)2]++C^N→[Fe(C^N)3]2++Cl−

Experimentally determined Δ*V* ^‡^ values indicate that the I_d_ mechanism is preferred for solvent exchange reactions in Fe­(II) complexes.^62^ Therefore, reactions 6, 7, 7, and 8 should be less competitive with reactions 2 and 5, respectively. These comparisons will be used to further test and validate the computational model employed in this work.

### d-Activated Dissociative Interchange Mechanism: Reaction Paths

#### First C^N Ligand Coordination

The potential energy surface for reaction 2 is shown in Figure 1. The associated energy barrier Δ*E* ^‡^ is 5.69 kcal/mol, and the I_d_ mechanism is evidenced by the premature release of the Cl^–^ anion when the Fe–C bond is not completely formed yet (∼4 Å, coordinate 18). The formation of the Fe–C bond releases a large amount of energy, making this process virtually irreversible. A relatively large energy barrier is required to coordinate the N atom, although much more favorable than the reverse reaction. In total, the reaction is exothermic with an associated Δ*E* of −20.2 kcal/mol. The reaction considering *cis*-[FeCl_2_(DMF)_4_], shown in Figure S2, exhibits a very similar profile.

**Figure 1:**
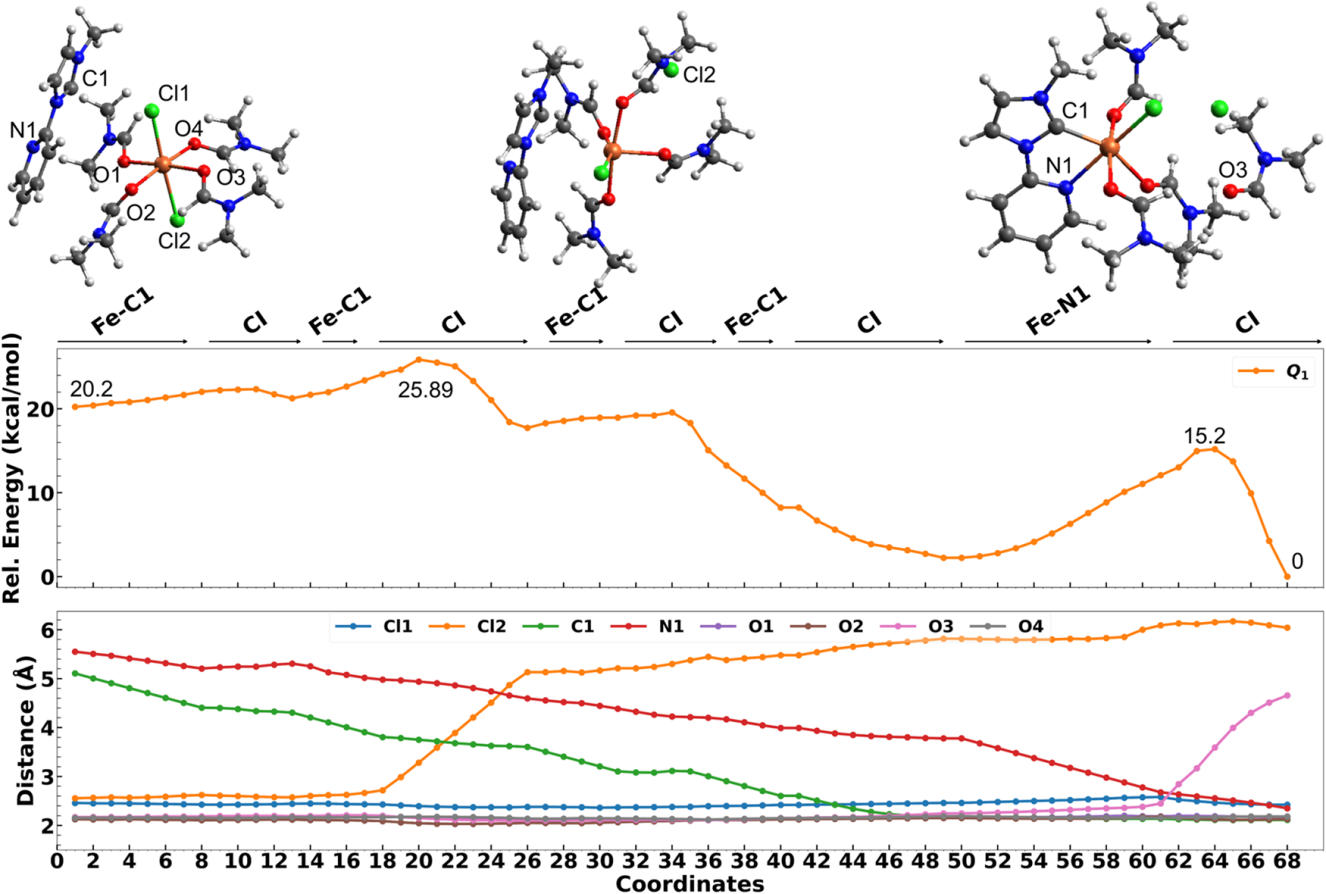
B3LYP/6–31+G­(d,p) energy profile for the *trans*-[FeCl_2_(DMF)_4_] + C^N → [FeCl­(C^N)­(DMF)_3_]^+^ + DMF + Cl^–^ reaction in the Q_1_ state. Fe–C1 and Fe–N1 indicate relaxed scan optimizations of the Fe–C1 and Fe–N1 distances, respectively. CI refers to coordinate interpolation. The bottom panel plots the distances between the Fe center and the indicated atom throughout the path. The XYZ coordinates can be downloaded from the article website.

#### Second C^N Ligand Coordination and Cl^–^ Recombination

The second C^N ligand can substitute two DMF units in the [FeCl­(C^N)­(DMF)_3_]^+^ intermediate through reaction 3 yielding [FeCl­(C^N)_2_(DMF)]^+^, as shown in Figure 2. The process is kinetically fast (Δ*E* ^‡^ = 5.02 kcal/mol) and highly exothermic (Δ*E* = −18.27 kcal/mol), thereby it must be considered mostly irreversible. The relative positions of the N,N and C,C pairs of coordinating atoms in [FeCl­(C^N)_2_(DMF)]^+^ are *cis*–*cis* for **A1**, *cis*–*trans* for **B1** and *trans*–*cis* for **C1** (Scheme 4), as in analogous [RuL_3_]^2+^ complexes.^78,79^ These species can quickly substitute the DMF ligand by a chloride anion (reaction 4) producing [FeCl_2_(C^N)_2_] as a more stable product, as shown in Figure 3, whereas the production of [Fe­(C^N)_2_(DMF)_2_]^+^ through reaction of [FeCl­(C^N)_2_(DMF)]^+^ with DMF is clearly unfavorable (see Figure S3). Therefore, the **A1**, **B1**, and **C1** structures shown in Scheme 4 correspond to the [FeCl_2_(C^N)_2_] species. Note that the formation of the *trans* intermediates **E1** and **F1** is very unlikely,^77^ and that only one optical enantiomer of each intermediate is considered since enantiomerism is not expected to alter the reactivity. The formation of **B1** and **C1** is reported in Figures S4 and S5 and exhibits a similar I_d_ mechanism than that of **A1** displayed in Figure 2, although with larger energy barriers (Δ*E* ^‡^ = 9.2 and 6.0 kcal/mol for **B1** and **C1**, respectively).

**Figure 2:**
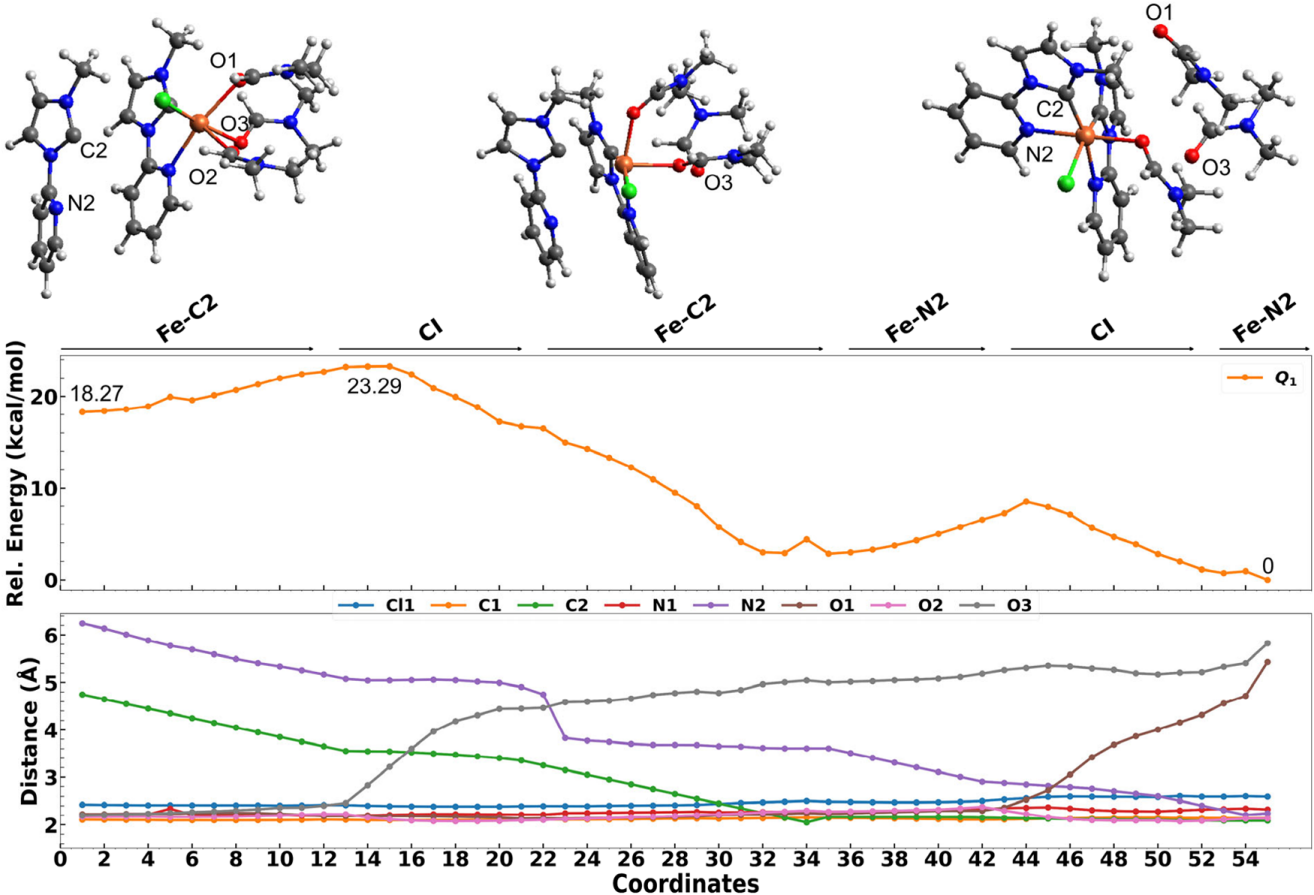
B3LYP/6–31+G­(d,p) energy profile for the [FeCl­(C^N)­(DMF)_3_]^+^ + C^N → [FeCl­(C^N)_2_DMF]^+^ (**A1**) + 2 DMF reaction in the Q_1_ state. Fe–C2 and Fe–N2 indicate relaxed scan optimizations of the Fe–C2 and Fe–N2 distances, respectively. CI refers to coordinate interpolation. The bottom panel plots the distances between the Fe center and the indicated atom throughout the path. The XYZ coordinates can be downloaded from the article website.

**Figure 3:**
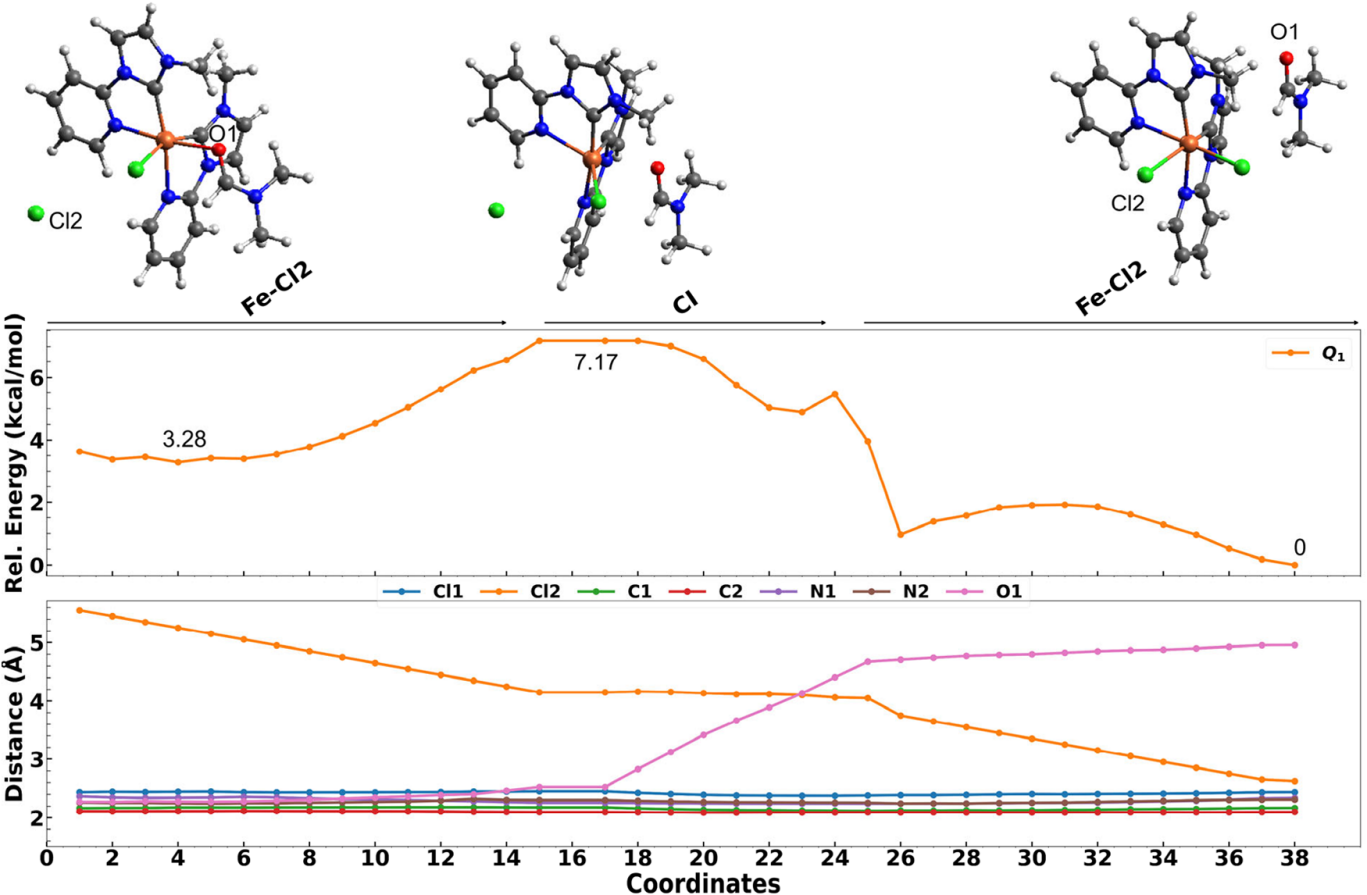
B3LYP/6–31+G­(d,p) energy profile for the [FeCl­(C^N)_2_DMF]^+^ + Cl^–^ → *cis*,*xxx*-[FeCl_2_(C^N)_2_] + DMF reaction in the quintet state (Q_1_). Fe–Cl2 indicates a relaxed scan optimization of the Fe–Cl2 distance, and CI refers to coordinate interpolation. The bottom panel plots the distances between the Fe center and the indicated atom throughout the path. The XYZ coordinates can be downloaded from the article website.

**Scheme 4:**
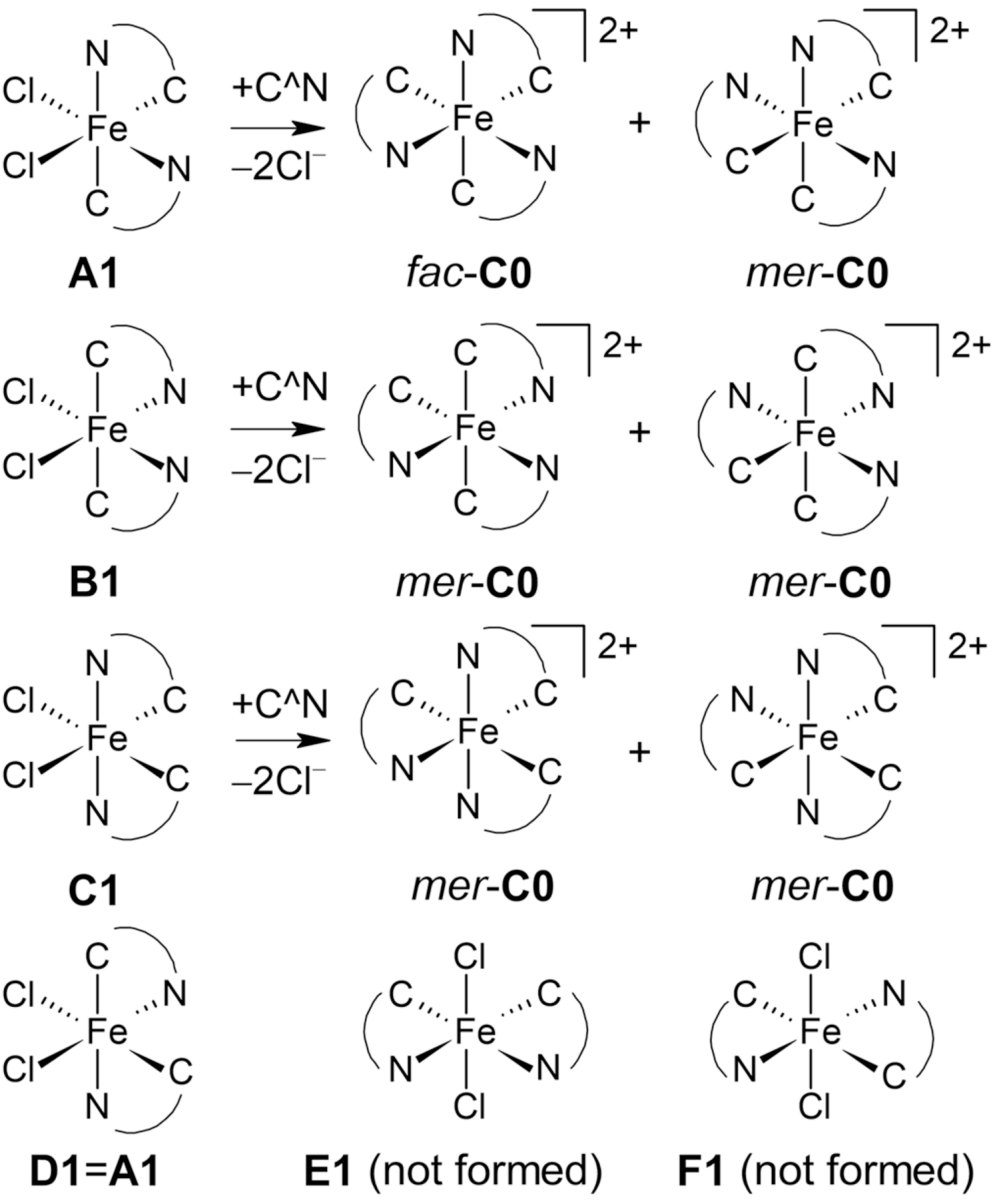
Stereochemistry of [FeCl_2_(C^N)_2_] Precursors and **C0** Final Complexes through reaction 3 ^a^

a Only one enantiomer is drawn, for the sake of clarity.

#### [FeCl_2_(C^N)_2_] Stabilities and Geometries

Considering that **B1** and **C1** can only yield the *mer* isomer whereas **A1** can produce both *mer* and *fac* isomers (Scheme 4),^78^ it becomes apparent that the relative abundance of the [FeCl_2_(C^N)_2_] stereoisomers plays a fundamental role in the *fac*/*mer* ratio of the final [Fe­(C^N)_3_]^2+^ species of reaction 5. Even though kinetic control seems to favor **A1** and **C1** over **B1** (Figures 2, S4 and S5), the analysis is based only on a few representative reaction paths that do not completely sample the whole reactive space. It is worth analyzing the **A1**/**B1**/**C1** thermodynamic stability to reinforce the prediction. The Gibbs energies at different electronic states is shown in Figure 4a. In all cases, the most stable electronic state is the first quintet state (Q_1_), indicating that the increased ligand field provided by the two neutral pyridyl–NHC ligands is not high enough to attain a low-spin configuration. Thus, further reactivity is considered to continue in the Q_1_ state as open-shell organometallics.^80^ Even though the most stable isomers are **A1** and **B1**, the formation of **A1** has the fastest kinetics and therefore it is deemed as the most abundant and therefore chemically relevant [FeCl_2_(C^N)_2_] intermediate among the three possibilities.

**Figure 4:**
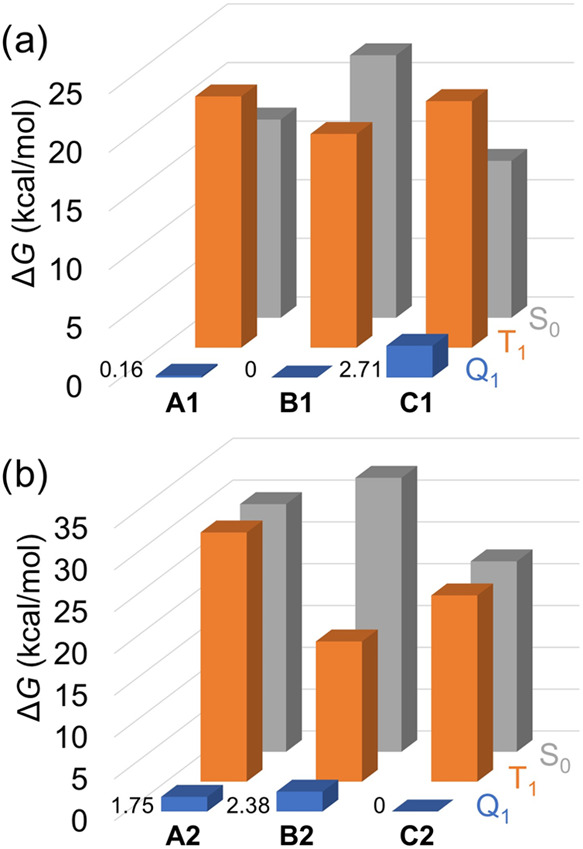
Δ*G* values for the [FeCl_2_(C^N)_2_] intermediates computed with respect to the most stable isomer in the Q_1_ state. Numerical data is summarized in Table S2. Geometries optimized with the B3LYP/6–31G* method and energies refined with the domain-based local pair natural orbital coupled cluster singles, doubles, and perturbative triples [DLPNO–CCSD­(T)] method. Thermal correction to Gibbs free energy computed at the B3LYP/6–31G* level of theory.

Witas et al.^52^ reported the formation of both **A1** and **C1** isomers in acetonitrile with a final 3:2 ratio after approximately 5 h of the reaction start. In this solvent, the present computational methodology indicates that increased ligand field produced by the ACN ligands leads to singlet low-spin (S_0_) complexes (Figure S6). Interestingly, **A1** and **C1** are predicted to be the most stable isomers, the two configurations detected in the experiments.^52^ Meggers and co-workers describe the formation of the **C1** isomer in a related Fe­(II) compound also in acetonitrile, although this preference is strongly directed by the π-stacking interaction of the mesityl groups.^81^

#### Third C^N Ligand Complexation

The production of **C0** via an I_d_ mechanism is given by reaction 5. Several chemical pathways can be considered for the formation of the Fe–C or Fe–N bonds and the coupled Cl^–^ release. The formation of the *mer* isomer is statistically more favorable since this label comprises several arrangements which can be mediated by all **A1**/**B1**/**C1** intermediates. According to Scheme 4, there is only one possibility to form *fac*-**C0** and it is mediated by **A1**. Consequently, this section studies in detail the production of the *fac*-**C0** isomer to serve as a mechanistic model and identify the electronic states and molecular motions involved in the process. Figure 5 shows the FAC-ID-1 path (reaction 5) in which a **L0** C^N ligand coordinates **A1** (**A1**→*fac*-**C0**). The formation of the N and C coordination bonds between the C^N ligand and Fe proceeds with an energy barrier of 1.74 kcal/mol in the Q_1_ state. Figure S7 shows an additional FAC-ID-2 path with a different C^N initial orientation, whereas Figure S8 displays the formation of *mer*-**C0** through the corresponding MER-ID-1 path. The activation energies for these latter pathways are slightly larger (Δ*E* ^‡^ = 4.9 and 2.9 kcal/mol for FAC-ID-2 and MER-ID-1, respectively) than that of FAC-ID-1 channel (Δ*E* ^‡^ = 1.74 kcal/mol).

**Figure 5:**
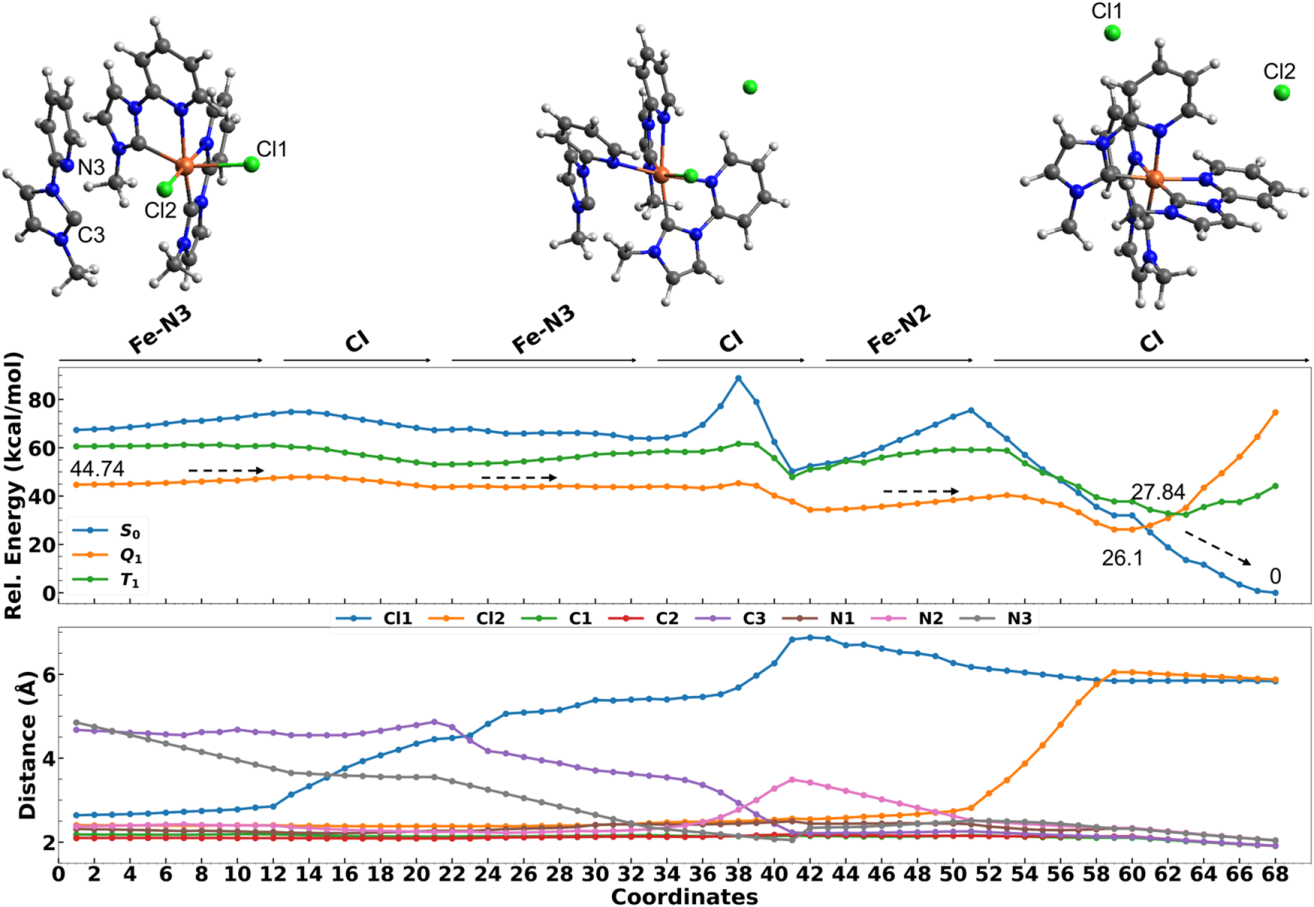
B3LYP/6–31+G­(d,p) complete energy profile for the FAC-ID-1 path yielding *fac*-**C0** through and I_d_ mechanism. The reaction corresponds to *cis*,*xxx*-[FeCl_2_(C^N)_2_] (**A1**) + C^N → *fac*-[Fe­(C^N)_3_]^2+^ + *mer*-[Fe­(C^N)_3_]^2+^ + 2 Cl^–^. Fe–N3 and Fe–N2 indicate relaxed scan optimizations of the Fe–N3 and Fe–N2 distances, respectively. CI refers to coordinate interpolation. The bottom panel plots the distances between the Fe center and the indicated atom throughout the path. The dotted arrows indicate the reaction path. The XYZ coordinates can be downloaded from the article website.

The closed-shell S_0_ state is populated only at the very last step of the process via accessible Q_1_/S_0_ crossings, after which all Fe–N and Fe–C bond lengths progress toward the typical equilibrium values for Fe­(II) complexes. The crossings obtained via coordinate interpolations are fairly good estimates of the minimum energy crossing points (MECPs), computed with ORCA 6.1 ^82^ and shown in Figure S9. The S_0_ – Q_1_ energy differences in the MECPs are smaller than in the estimated crossings due to the energy degeneracy imposed in the crossing minimizations, absent in the coordinate interpolations. The Q_1_ energy is slightly larger (≤2 kcal/mol) in the MECP structures as compared to the estimated crossing points, and in one case (FAC-D-1 path) the optimization stabilizes the Q_1_ state about 0.5 kcal/mol. The root mean square deviation (RMSD) values computed between both estimated and optimized crossing point structures are very small (Figure S9). These results confirm that the MECP structures are accessible across the proposed mechanisms. Likely, the triplet T_1_ state acts as an intermediate state since the coupling between S_0_ and Q_1_ is only of second order.^83^ Intersystem crossing in this region is assumed based on the energy difference between states of different multiplicity and the well-known low spin (S_0_) of the final complex.

Globally, the FAC-ID-1, FAC-ID-2, and MER-ID-1 mechanisms exhibit similar features. The representative reaction pathways computed in this work do not allow a quantitative prediction of the *fac*/*mer* ratios based on activation barriers, which require an exhaustive exploration of the conformational reactive space likely possible only via *ab initio* molecular dynamics. Instead, this contribution focuses on establishing the main features (molecular motions, electronic energies, and spin multiplicities) of the molecular pathways that lead to the formation of the **C0** complex, starting from the FeCl_2_ and the NHC reagents.

All in all, it becomes apparent that the proposed mechanism for reaction 5 is kinetically feasible at room temperature and strongly irreversible, since ca. 40 kcal/mol are released because of the C and N coordination and the Cl^–^ release.

### Dissociative Mechanism: Reaction Paths

#### Cl^–^/DMF Dissociation and First and Second C^N Complexation

In the purely dissociative D mechanism, the rate limiting step is the bond breaking and ligand release to produce a species with a decreased coordination number.^62^ The capacities of Cl^–^ or DMF to act as leaving groups in the *trans*-[FeCl_2_(DMF)_4_] species (reactions 6 and 7) are evaluated in Figure S10. The corresponding DFT relaxed scan profiles evidence that both leaving groups have comparable Δ*E* ^‡^ values between them (5.07 and 5.38 kcal/mol for DMF and Cl^–^, respectively) and with reaction 2 (5.69 kcal/mol, Figure 1), even though the dissociative cleavages are endothermic and thus more reversible. Therefore, even though the energy barriers are comparable between the I_d_ and D mechanisms, the reversibility of reactions 6 and 7 suggests that the former is more competitive. DMF is a slightly better leaving group than Cl^–^ since [FeCl_2_(DMF)_3_] is more stable than [FeCl­(DMF)_4_]^+^ with respect to the corresponding reactants.

Reaction 8 corresponds to the C^N coordination to the Fe center with a decreased coordination number and is considered barrierless (see the analogous reaction 13 below). Reactions 9 and 10, which corresponds to a second Fe–O or Fe–Cl bond break, are studied in Figure S11. Clearly, the Fe–O scission (Δ*E* ∼ 1 and Δ*E* ^‡^ ∼ 4 kcal/mol) is more competitive than the Fe–Cl cleavage (Δ*E* ∼ 5 and Δ*E* ^‡^ > 10 kcal/mol) both from a kinetic and thermodynamic perspective, and the former is thus considered the preferred pathway. The addition of a second NHC ligand (reaction 11) is also considered to be barrierless, followed by the favorable reaction 4 (see Figure 3), in which the intermediates **A1**, **B1**, and **C1** are formed.

#### [FeCl_2_(C^N)_2_] Chloride Loss

Figure S12 studies the Cl^–^ anion release prior to the reaction with the C^N moiety (reaction 12). Activation energies for these processes are about 3 and 5 kcal/mol for **A1** and **B1**, respectively, which are relatively low values thermally accessible at room temperature. **A2**, **B2**, and **C2** display bipyramidal trigonal geometries (Figure S12) and **C2** is the most stable isomer among them (Figure 4b). **A2** and **B2** abundance is expected to be limited since the recombination with Cl^–^ is more favorable (**A1** and **B1** are more stable than **A2** and **B2**, respectively), and reaction 5 can be kinetically more competitive (Δ*E* ^‡^ as low as 1.74 kcal/mol). Regarding **C2**, its formation from **C1** is favored, although the low stability of **C1** (Figure 4) questions the relevance of this path. Loss of the remaining Cl^–^ ligand through Fe–Cl dissociation, i.e., reaction 14, is characterized in Figure S13 and deemed very slow and thus not competitive, since activation energies exceed 13 kcal/mol.

12 [FeCl(C^N)2]+→[Fe(C^N)2]2++Cl−

Unimolecular **B2** ↔ **A2** ↔ **C2** isomerizations are also possible (Figure S14) with associated energy penalties ≤4.35 kcal/mol for **A2** ↔ **B2** and ≤ 7.10 kcal/mol for **B2** ↔ **C2**. These conversions compete with reaction 13, which is barrierless (although bimolecular). Therefore, some **A2** ↔ **B2** equilibration could be possible, depending on the concentration of the NHC ligand in the reaction microenvironment. The dynamic interconversion between intermediates could influence the final *fac*/*mer* ratios.

#### Third NHC Ligand Complexation

The last step in the formation of **C0** through a D mechanism is reaction 13. The coordination of the C^N ligand to the [FeCl­(C^N)_2_]^+^ intermediates **A2**, **B2**, and **C2** offer multiple possibilities. In this work, the C^N ligand is initially oriented toward the molecular face that contains the Fe–Cl bond, the less crowded molecular region, thereby considered as the preferred coordination site.

Figure 6 represents an **A2**→*fac*-**C0** path (FAC-D-1) in which the N6 and C5 atoms of **L0** bind Fe almost synchronously (coordinates 1–20), breaking the Fe–N3 linkage. Energetically, the path is almost planar in the Q_1_ state (the negative slope is very small) and connects with the crossing between the three S_0_, T_1_ and Q_1_ states. No significant energy barriers are predicted in this process. After population of S_0_ (likely via the T_1_ state),^83^ the molecule evolves toward the formation of *fac*-**C0** with an energy barrier of 7.3 kcal/mol through the formation of the Fe–N3 bond and the scission of the Fe–Cl connection (coordinates 30–50). Globally, the production of the low spin [Fe­(C^N)_3_]^2+^ complex via reaction 8 is highly exothermic (Δ*E ca.* −40 kcal/mol), thus considered irreversible. Reactions of the [FeCl­(C^N)_2_]^+^ intermediates **B2** and **C2** with **L0** should be considered to proceed analogously to those of **A2**, even though only the *mer* disposition is formed in these cases.

**Figure 6:**
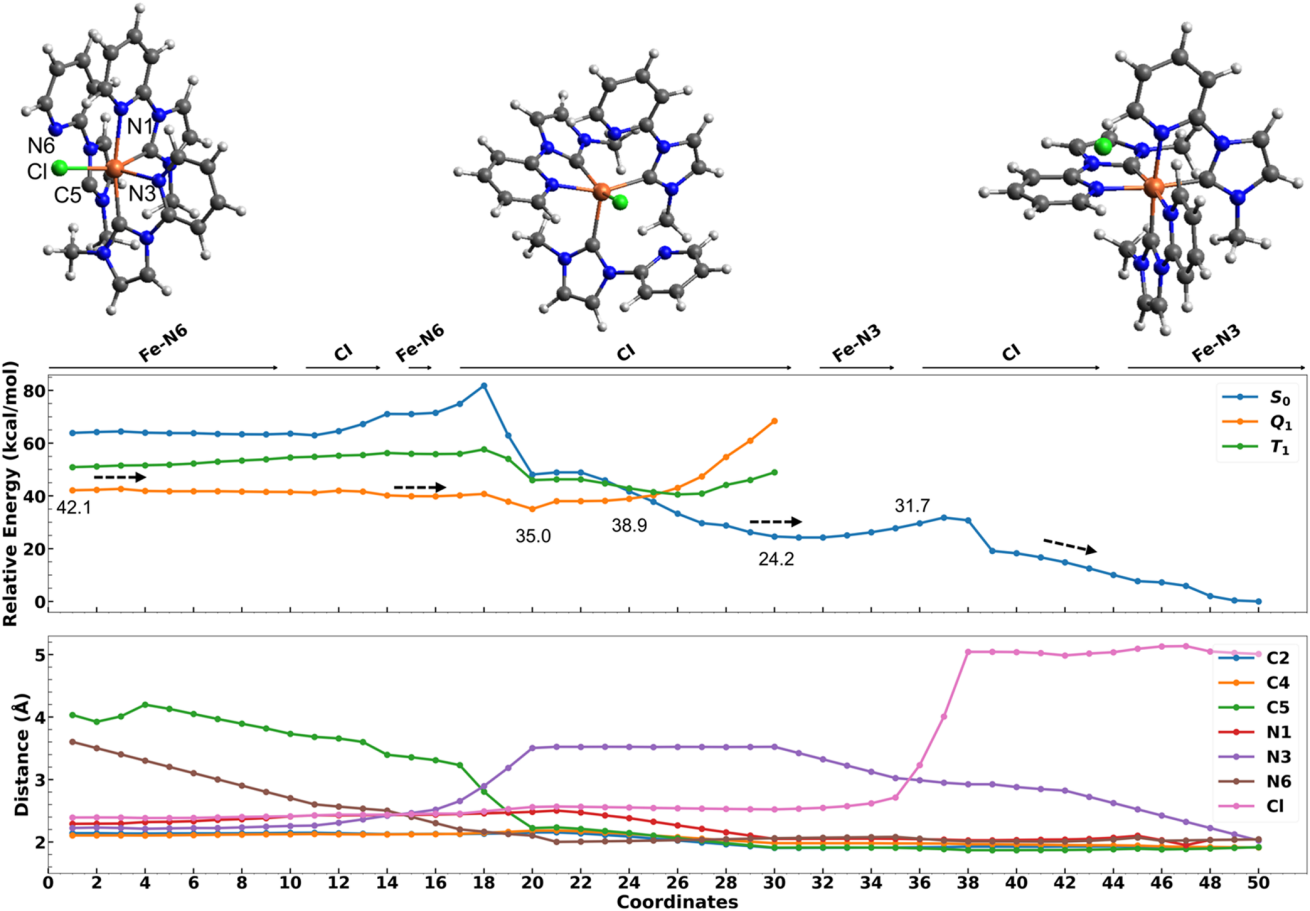
B3LYP/6–31+G­(d,p) complete energy profile for the FAC-D-1 path, yielding *fac*-**C0** through the reaction [FeCl­(C^N)_2_]^+^ + C^N → [Fe­(C^N)_3_]^2+^ + Cl^–^. Profiles obtained through combinations of relaxed scan calculations of Fe–N6 (in the Q_1_ state) and Fe–N3 (in the S_0_ state) bonds, and coordinate interpolations (CI). The bottom panel plots the distances between the Fe center and the indicated atom throughout the path. The dotted arrows indicate the reaction path.

Lastly, Figure S15 displays the energy profiles for the FAC-D-2 path. For both *fac* paths, the complexation of the third **L0** C^N ligand in the Q_1_ state breaks another Fe–N bond, which can be restored only if the system hops to the singlet ground state S_0_.

### I_d_ vs D Competition and Global Mechanistic Description

This section summarizes the mechanism of the **C0** formation starting from FeCl_2_ in DMF (Scheme 1), a representative synthesis of a Fe­(II)-C^N family of complexes of great scientific interest in the last years,^40,41,47,50,52,53,56,57,60^ and assesses the competition between I_d_ and D mechanisms^62^ and their intersections throughout the coordination process.

The main findings of this work are summarized in Figure 7. The coordination of the first C^N ligand through an I_d_ mechanism (reaction 2) has an estimated activation energy of 5.7 kcal/mol, similar to the D channel represented by reaction 6 followed by reaction 8, which is of 5.1–5.4 kcal/mol (corresponding to reactions 6 and 7, reaction 8 is expected to be barrierless). Therefore, both I_d_ and D mechanisms are kinetically competitive, even though the lifetime of [FeCl_2_(DMF)_3_] or [FeCl­(DMF)_4_]^+^ is expected to be short given the reversibility of the reaction displayed in Figure S10, which is endothermic. In any case, it is reasonable to conclude that [FeCl­(C^N)­(DMF)_3_]^+^ is the product of both mechanisms.

**Figure 7:**
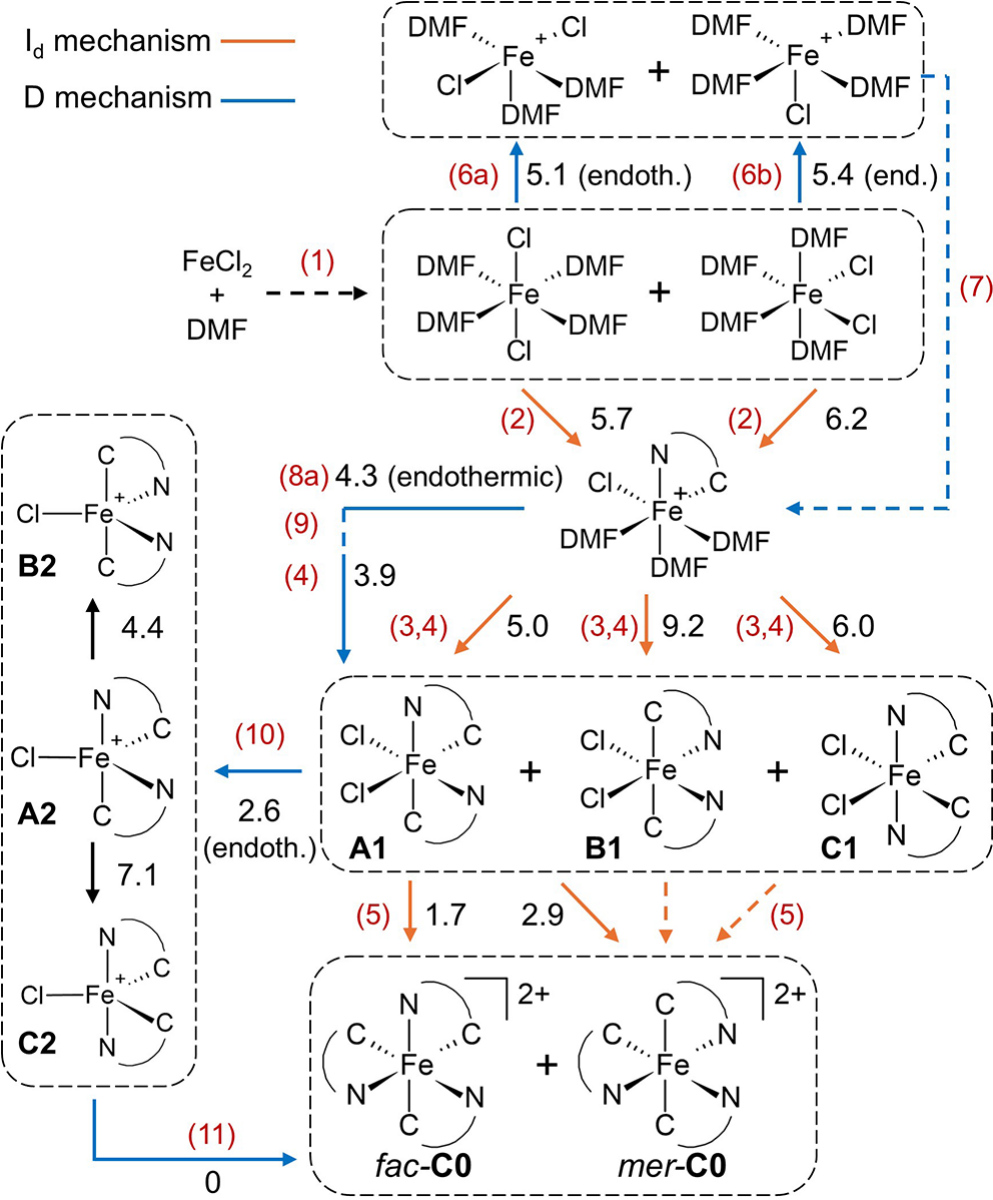
[Fe­(pyIm)_3_]^2+^ (**C0**) synthesis mechanism based on the present results. Reactions represented with solid arrows have been examined in this work, whereas dashed arrows indicate not studied reactions. Reaction numbers are shown within parentheses in dark red. Values next to the arrows indicate the Δ*E* ^‡^ values computed in this work (in kcal/mol).

[FeCl­(C^N)­(DMF)_3_]^+^ reacts with a second **L0** ligand via an I_d_ mechanism (reaction 3, activation energy 5 kcal/mol) to produce [FeCl­(C^N)_2_(DMF)]^+^ in the **A1** configuration (Figure 7). The formation of the **B1** and **C1** isomers is slower. On the other hand, the Fe–O scission corresponding to the D mechanism is slightly faster (activation energy of 4.3 kcal/mol) than reaction 3 (activation energy of 5.0 kcal/mol), although the D process is, again, endothermic.

The replacement in [FeCl­(C^N)_2_(DMF)]^+^ of DMF by a Cl^–^ anion (reaction 4) is favorable (activation energy of 3.9 kcal/mol and exothermic) and leads to the formation of the [FeCl_2_(C^N)_2_] intermediates **A1**, **B1**, and **C1** (Figure 7). The I_d_ mechanism directly coordinates the third C^N **L0** ligand through reaction 5 with an estimated energy barrier as low as 1.7 kcal/mol, whereas the D mechanism represented by reaction 12 exhibits an activation energy of 2.6 kcal/mol and is endothermic (Figure S12), deeming the I_d_ mechanism as kinetically more competitive in this step.

The global analysis of the C^N coordination energetics reveals that the complexation is stepwise, mostly occurs in the Q_1_ state, and that it is highly exothermic and thus irreversible. Kinetically, the D mechanism is more competitive in the first and second C^N coordinations, whereas the I_d_ mechanism is favored in the third one. Nevertheless, energy differences between the key steps are modest, and the static computational approach does not allow for the exploration of the complete reactive spaces. On the contrary, the I_d_ paths are the most favorable channels from a thermodynamic standpoint. Overall, these findings explain the large stability of these compounds and questions *fac* ↔ *mer* isomerizations, at least without a catalyst and within relatively short reaction times.

## Conclusions

The present study resolves the coordination mechanism of *N-*heterocyclic carbenes (NHC) of C^N type to Fe­(II) atoms to produce octahedral [Fe­(C^N)_3_]^2+^ complexes^40,41,47,50,52,53,56,57,60^ at the molecular and electronic levels using the complex [Fe­(pyIm)_3_]^2+^ (**C0**) as model case. A general theoretical protocol applicable to the study of other transition metal complexes is established and validated with available experimental evidence. Results confirm that the coordination of the three NHC ligands to the Fe center is sequential, in which the configuration and abundance of the [FeCl_2_(C^N)_2_] intermediates play a crucial role in dictating the final *fac* or *mer* isomerism of the [Fe­(C^N)_3_]^2+^ octahedral complex. The C^N coordination to the iron center is highly exothermic and it is considered irreversible, at least at short times in absence of a catalyst. Almost all reactivity takes place in the first quintet state Q_1_ (high spin molecules), since the singlet ground state S_0_ is only populated right before the formation of the last Fe–N coordination bond. Among the two mechanistic possibilities inferred from kinetic experiments at different pressures,^62^ namely the dissociative (D) and the d-activated dissociative interchange (I_d_) mechanisms, both are, in general, competitive. The former is kinetically more favorable for the first and second C^N coordination, whereas the I_d_ mechanism is faster for the third one. From a thermodynamic point of view, the I_d_ channel is preferred in all steps. Overall, these findings support that bond breaking contributes the most to the transition states, coherently with the lack of dependence between the ligand exchange kinetics and the ligand nature observed in the experiments.^62^

The dissociative mechanisms described in this study involve intrinsically little, or very little, dependence on the nature of the ligand in the exchange kinetics. However, more complex and sophisticated ligand designs^40,41,50,53,56,81^ can enable additional covalent and noncovalent interactions and steric hindrances absent in the pyIm structure considered in this work. In these cases, we hypothesize that energy profiles associated with the C^N ligand coordinations do highly depend on the ligand nature, and that a detailed comparison shall reveal the intrinsic competition that dictates the *fac*/*mer* abundance of the final octahedral complexes. Our laboratory is currently working in that direction.

## Computational Methods

### Geometry Optimizations and Reaction Energy Profiles

Unless otherwise stated, all geometry optimizations have been performed with the popular B3LYP functional in association with the 6–31G* basis set, as implemented in the Gaussian 16 software,^84^ using default settings and without imposing symmetry restrains. This level of theory provides good descriptions of the geometries of these types of systems, as repeatedly shown for related Fe­(II) systems.^4,44,46,50,56,57,85–87^ Optimized geometries were ensured to be true minima by computing the vibrational normal modes and confirming the absence of any imaginary frequency. The rigid-harmonic oscillator-ideal gas approximation has been used to compute Gibbs corrections to electronic energies at 298.15 K and 1 atm.^88^ Solvent effects (DMF) have been described with the polarizable continuum model (PCM)^89^ using the Gaussian 16 default settings. Empirical corrections have been incorporated by means of the Grimme D3 method.^90^ All reaction paths have been computed with the B3LYP/6–31+G­(d,p) basis set with single point calculations on top of the converged B3LYP/6–31G* geometries. The 6–31+G­(d,p) basis set already provides converged results with respect to the larger 6–311++G­(2df,2pd) basis set, as shown in Figure S16 for reaction 2. MECPs between the Q_1_ and S_0_ states have been optimized with ORCA 6.1 ^82^ and the B3LYP/G/6–31G* basis set in DMF.

### DFT and Coupled Cluster Benchmark

The DFT/B3LYP/6–31G* method is validated by computing the Q_1_ energy profile of the Fe–C5 and Fe–N6 bond formation in the path FAC-D-1 (Figure 6) with other functionals and the same basis set on top of the DFT/B3LYP/6–31G* optimized geometries (Figure S17). The chosen functionals and the percentage of nonlocal Hartree–Fock exchange (X) comprise the global-hybrid GGA functionals B3LYP* (X = 15%),^91,92^ B3LYP (X = 20%),^93–95^ PBE0 (X = 25%),^96^ M06 (X = 27%),^97^ the range-separated hybrid GGA + molecular mechanics functional ωB97X-D (X = 22 to 100%, depending on the distance range),^98^ and the global-hybrid meta-NGA functional MN15 (X = 44%).^99^ In addition, the same profile has been computed with the wave function method domain-based local pair natural orbital coupled cluster singles, doubles, and perturbative triples DLPNO–CCSD­(T)^100^ in combination with the def2-TZVP basis set, as implemented in ORCA 6.^82^ The def2-TZVP/C auxiliary basis set was used throughout all coupled-cluster computations. Solvent effects (DMF) were included through conductor-like polarizable continuum model (CPCM)^101,102^ using the default settings. Figure S17 shows that all employed electronic structure methods provide fully consistent energy profiles, with small energy differences that do not alter either the mechanistic description or the conclusions presented in this work in any case.

### Spin State Ordering

Figure S18 compares the S_0_, T_1_, and Q_1_ energies on top of the Q_1_ optimized geometry of the reactants of reaction 2 (Figure 1) computed with the B3LYP, B3LYP*, and TPSSh functionals and the 6–31G* basis set. The B3LYP* and TPSSh functionals provide correct spin state ordering compared to reference CASPT2 ^103^ or experimental^91^ results for related Fe­(II) complexes. The spin-state transition, i.e., the energy gap between the minima of the lowest singlet and quintet potential wells (Δ*E*_HL_ = *E*_HS_ – *E*_LS_), for both *fac* **-C0** and *mer*-**C0** compounds, is also computed with the three functionals. Geometry optimizations were performed with the 6–31G* basis set, whereas final energies were refined with the 6–31+G­(d,p) basis set. The ordering of the states of different multiplicities provided by B3LYP are consistent with those of B3LYP* and TPSSh functionals, thus validating the B3LYP description.

### Relaxed Scans

Reaction pathways have been obtained by means of systematic relaxed scans of reaction coordinates at the same level of theory as the geometry optimizations described above, using the Gaussian 16 software. The studied reaction coordinates and the obtained data points are indicated in the corresponding figures. Unless otherwise stated, step sizes of 0.1 Å and 5° have been employed for bond distances and angles, respectively. The geometry optimization convergence threshold for the root-mean-square of the first energy derivative with respect to the coordinates was set to 10^–3^ Hartree/Bohr. Note that, in relaxed scans, all molecular parameters are fully optimized at each step except the reaction coordinate, which is deliberately fixed each step to be progressively scanned, providing satisfactory estimations of reaction paths. This method is particularly useful to describe the numerous and complex potential energy surfaces (PES) involved in the synthesis mechanism of [Fe­(C^N)_3_]^2+^, where the relatively large molecular size and multiple coordination sites make the optimization of transition states intricate and unmanageable.

### Coordinate Interpolations

Relaxed scans often suffer from hysteresis, i.e., relatively large and abrupt topological changes between two relaxed scan steps that produce unconnected paths. In addition, certain PES areas exhibit poor geometry convergence. The connection between all points represented in the reaction paths is ensured through coordinate interpolations (CI). Seven intermediate geometries have been generated between two converged geometries along the reaction path not connected anymore due to hysteresis/convergence failure of one or more consecutive relaxed scan steps, to ensure connected and thus chemically meaningful pathways. Interpolated coordinates were calculated using the geodesic interpolation method developed by Martínez and co-workers.^104^

### Nudged Elastic Band

To validate the calculation of reaction profiles with relaxed scans and coordinate interpolations, the reaction 2 profile (Figure 1) was also computed with the nudge elastic band (NEB) method as implemented in ORCA 6.1.^82^ This method provides the minimum energy path (MEP) between reactants and products, which are provided by the user as guess geometries. Two MEPs have been computed, one for the Fe–C1 bond formation and another one for the Fe–N1 coordination, using the B3LYP/G functional and the 6–31G* basis set, including empirical dispersion corrections. Solvent effects (DMF) were incorporated using the CPCM model. Two different convergence thresholds, default (Tol_MaxFP_I = 10^–3^ and Tol_RMSFP_I = 5·10^–4^ Eh/Bohr) and tight (Tol_MaxFP_I = 5·10^–4^ and Tol_RMSFP_I = 2.5·10^–4^ Eh/Bohr), have been used. Final energies in both cases are computed with the B3LYP/6–31+G­(d,p) method making use of the Gaussian 16 program. Results are shown in Figure S19. The comparison of the MEP maxima and minima with those of the relaxed scan + coordinate interpolation strategy reveals very similar energy barriers for both Fe–N and Fe–C bond formations, differing in less than 2 kcal/mol. A difference of 3.5 kcal/mol is found for the relative energy of the reactants (coordinate 1) with respect to the products (energy = 0), not changing in any case the description of the reaction and the scientific implications. This difference is very reasonable considering the differences between the methodologies used to compute panels a and b. The resultant geometries of both methods indicate that the two pathways are almost equivalent and involve the same molecular motions. The energy profile shapes change because the size of the nuclear displacements is inherently different, but both mechanisms are equivalent. The XYZ geometries of both paths are provided as electronic Supporting Information.

### Thermal and Entropy Contributions

It is expected that the entropy contribution is similar for all reaction paths. Since no TSs have been optimized here, Gibbs energies can only be calculated for the optimized reactants and products. Table S3 compiles the Δ*G* values at 298 K for the *fac* and *mer* paths considered in this work to quantify the thermal and entropic contributions between products and reactants. All pathways remain strongly exergonic independently of the considered thermodynamic potential. The Δ*G* – Δ*E* values are very similar for all paths, ranging from 6 to 8.5 kcal/mol in all cases, validating the hypothesis that the entropy contribution is similar for all reaction paths. Nonetheless, these values must be seen as upper bounds of the entropy contribution in the TS-like regions far from the product formation (e.g., Figures 2 and 3) or in flat profiles (e.g., Figures 5 and 6), where the covalent bonding is not significantly altered yet, and the entropy variation is expected to be low.

## Supplementary Material





## Notes

Additional reaction paths, relative Gibbs energy values, MEP profiles, MECP optimizations, spin state ordering, additional thermodynamic data, basis set effect analysis, B3LYP validation, experimental UV–vis spectrum of FeCl_2_ and **LH0** in DMF (PDF)
Cartesian coordinates of the reaction paths (ZIP)

## Notes

Department of Chemical Sciences, University of Padova, Via Marzolo 1, 35121 Padova, Italy

## References

[1] Yun B.-S., Kim S.-Y., Kim J.-H., Son H.-J., Kang S. O. (2021). Homoleptic
Cyclometalated Dibenzothiophene–NHC–Iridium­(Iii) Complexes
for Efficient Blue Phosphorescent Organic Light-Emitting Diodes. J. Mater. Chem. C.

[2] Cooke G., Máille G. M. Ó., Quesada R., Wang L., Varughese S., Draper S. M. (2011). Substituted Pyridazines as Ligands
in Homoleptic (Fac and Mer) and Heteroleptic Ru­(II) Complexes. Dalton Trans..

[3] Lathion T., Guénée L., Besnard C., Bousseksou A., Piguet C. (2018). Deciphering the Influence of Meridional versus Facial
Isomers in Spin Crossover Complexes. Chem. -
Eur. J..

[4] Magra K., Francés-Monerris A., Cebrián C., Monari A., Haacke S., Gros P. C. (2022). Bidentate Pyridyl-NHC
Ligands: Synthesis, Ground and Excited State Properties of Their Iron­(II)
Complexes and the Role of the Fac/Mer Isomerism. Eur. J. Inorg. Chem..

[5] Chandrasekhar, V. ; Pointillart, F. . Organometallic Magnets; Springer, 2019.

[6] Balzani, V. ; Ceroni, P. ; Juris, A. Photochemistry and Photophysics: Concepts, Research, Applications; John Wiley & Sons, 2014.

[7] Lee J., Chen H.-F., Batagoda T., Coburn C., Djurovich P. I., Thompson M. E., Forrest S. R. (2016). Deep Blue Phosphorescent Organic
Light-Emitting Diodes with Very High Brightness and Efficiency. Nat. Mater..

[8] Yersin, H. Highly Efficient OLEDs with Phosphorescent Materials; John Wiley & Sons, 2008.

[9] Costa, R. D. Light-Emitting Electrochemical Cells. In Concepts, Advances and Challenges; Springer, 2017; Vol. 1.

[10] Fusi G. M., Gazzola S., Piarulli U. (2022). Chiral Iron Complexes in Asymmetric
Organic Transformations. Adv. Synth. Catal..

[11] de
Groot L. H. M., Ilic A., Schwarz J., Wärnmark K. (2023). Iron Photoredox
Catalysis–Past, Present, and Future. J. Am. Chem. Soc..

[12] Dalle K. E., Warnan J., Leung J. J., Reuillard B., Karmel I. S., Reisner E. (2019). Electro- and Solar-Driven
Fuel Synthesis
with First Row Transition Metal Complexes. Chem.
Rev..

[13] Kim D., Dang V. Q., Teets T. S. (2023). Improved Transition Metal Photosensitizers
to Drive Advances in Photocatalysis. Chem. Sci..

[14] Prier C. K., Rankic D. A., MacMillan D. W. C. (2013). Visible
Light Photoredox Catalysis
with Transition Metal Complexes: Applications in Organic Synthesis. Chem. Rev..

[15] Ehnbom A., Ghosh S. K., Lewis K. G., Gladysz J. A. (2016). Octahedral Werner
Complexes with Substituted Ethylenediamine Ligands: A Stereochemical
Primer for a Historic Series of Compounds Now Emerging as a Modern
Family of Catalysts. Chem. Soc. Rev..

[16] McKenzie L.
K., Bryant H. E., Weinstein J. A. (2019). Transition Metal Complexes as Photosensitisers
in One- and Two-Photon Photodynamic Therapy. Coord. Chem. Rev..

[17] Monro S., Colón K. L., Yin H., Roque J., Konda P., Gujar S., Thummel R. P., Lilge L., Cameron C. G., McFarland S. A. (2019). Transition Metal Complexes and Photodynamic
Therapy
from a Tumor-Centered Approach: Challenges, Opportunities, and Highlights
from the Development of TLD1433. Chem. Rev..

[18] Cutler C. S., Hennkens H. M., Sisay N., Huclier-Markai S., Jurisson S. S. (2013). Radiometals for Combined Imaging and Therapy. Chem. Rev..

[19] Yusoh N. A., Gill M. R., Tian X. (2025). Advancing Super-Resolution Microscopy
with Metal Complexes: Functional Imaging Agents for Nanoscale Visualization. Chem. Soc. Rev..

[20] Chábera P., Lindh L., Rosemann N. W., Prakash O., Uhlig J., Yartsev A., Wärnmark K., Sundström V., Persson P. (2021). Photofunctionality of Iron­(III) N-Heterocyclic
Carbenes
and Related d^5^ Transition Metal Complexes. Coord. Chem. Rev..

[21] Wegeberg C., Wenger O. S. (2021). Luminescent First-Row Transition
Metal Complexes. JACS Au.

[22] Sinha N., Wenger O. S. (2023). Photoactive Metal-to-Ligand
Charge Transfer Excited
States in 3d6 Complexes with Cr0, MnI, FeII, and CoIII. J. Am. Chem. Soc..

[23] Chan A. Y., Ghosh A., Yarranton J. T., Twilton J., Jin J., Arias-Rotondo D. M., Sakai H. A., McCusker J. K., MacMillan D. W. C. (2023). Exploiting
the Marcus Inverted Region for First-Row Transition Metal–Based
Photoredox Catalysis. Science.

[24] Jiang T., Bai Y., Zhang P., Han Q., Mitzi D. B., Therien M. J. (2020). Electronic
Structure and Photophysics of a Supermolecular Iron Complex Having
a Long MLCT-State Lifetime and Panchromatic Absorption. Proc. Natl. Acad. Sci. U.S.A..

[25] Wenger O. S. (2018). Photoactive
Complexes with Earth-Abundant Metals. J. Am.
Chem. Soc..

[26] Kjær K. S., Kaul N., Prakash O., Chábera P., Rosemann N. W., Honarfar A., Gordivska O., Fredin L. A., Bergquist K. E., Häggström L., Ericsson T., Lindh L., Yartsev A., Styring S., Huang P., Uhlig J., Bendix J., Strand D., Sundström V., Persson P., Lomoth R., Wärnmark K. (2019). Luminescence
and Reactivity of a Charge-Transfer Excited Iron Complex with Nanosecond
Lifetime. Science.

[27] Morgan K., Lidin S., Styring S., Essén S., Persson P., Honarfar A., Uhlig J., Ericsson T., Bendix J., Thyrhaug E., Schnadt J., Wärnmark K., Sobkowiak A., Prakash O., Nahhas A. El., Liu Y., Chábera P., Tatsuno H., Lomoth R., Fredin L. A., Ericson F., Häggström L., Kjær K. S., Handrup K., Huang P., Sundström V., Harlang T. C. B. (2017). A Low-Spin Fe­(III) Complex with 100-Ps Ligand-to-Metal
Charge Transfer Photoluminescence. Nature.

[28] Zhang W., Kjær K. S., Alonso-Mori R., Bergmann U., Chollet M., Fredin L. A., Hadt R. G., Hartsock R. W., Harlang T., Kroll T., Kubiček K., Lemke H. T., Liang H. W., Liu Y., Nielsen M. M., Persson P., Robinson J. S., Solomon E. I., Sun Z., Sokaras D., van Driel T. B., Weng T.-C., Zhu D., Wärnmark K., Sundström V., Gaffney K. J. (2017). Manipulating Charge
Transfer Excited State Relaxation and Spin Crossover in Iron Coordination
Complexes with Ligand Substitution. Chem. Sci..

[29] Herr P., Kerzig C., Larsen C. B., Häussinger D., Wenger O. S. (2021). Manganese­(I) Complexes with Metal-to-Ligand
Charge
Transfer Luminescence and Photoreactivity. Nat.
Chem..

[30] Sinha N., Pfund B., Wegeberg C., Prescimone A., Wenger O. S. (2022). Cobalt­(III) Carbene Complex with an Electronic Excited-State
Structure Similar to Cyclometalated Iridium­(III) Compounds. J. Am. Chem. Soc..

[31] Förster C., Heinze K. (2022). Bimolecular Reactivity
of 3d Metal-Centered Excited
States (Cr, Mn, Fe, Co). Chem. Phys. Rev..

[32] Bizzarri C., Spuling E., Knoll D. M., Volz D., Bräse S. (2018). Sustainable
Metal Complexes for Organic Light-Emitting Diodes (OLEDs). Coord. Chem. Rev..

[33] Wenger O. S. (2019). Is Iron
the New Ruthenium?. Chem. - Eur. J..

[34] Dierks P., Vukadinovic Y., Bauer M. (2022). Photoactive Iron Complexes:
More
Sustainable, but Still a Challenge. Inorg. Chem.
Front..

[35] Liu Y., Persson P., Sundström V., Wärnmark K. (2016). Fe N-Heterocyclic
Carbene Complexes as Promising Photosensitizers. Acc. Chem. Res..

[36] Auböck G., Chergui M. (2015). Sub-50-Fs Photoinduced
Spin Crossover in [Fe­(Bpy)_3_]^2+^. Nat. Chem..

[37] Zhang W., Alonso-Mori R., Bergmann U., Bressler C., Chollet M., Galler A., Gawelda W., Hadt R. G., Hartsock R. W., Kroll T., Kjær K. S., Kubiček K., Lemke H. T., Liang H. W., Meyer D. A., Nielsen M. M., Purser C., Robinson J. S., Solomon E. I., Sun Z., Sokaras D., van Driel T. B., Vankó G., Weng T.-C., Zhu D., Gaffney K. J. (2014). Tracking Excited-State
Charge and Spin Dynamics in Iron Coordination Complexes. Nature.

[38] Kjær K. S., Van Driel T. B., Harlang T. C. B., Kunnus K., Biasin E., Ledbetter K., Hartsock R. W., Reinhard M. E., Koroidov S., Li L., Laursen M. G., Hansen F. B., Vester P., Christensen M., Haldrup K., Nielsen M. M., Dohn A. O., Pápai M. I., Møller K. B., Chabera P., Liu Y., Tatsuno H., Timm C., Jarenmark M., Uhlig J., Sundstöm V., Wärnmark K., Persson P., Németh Z., Szemes D. S., Bajnóczi É., Vankó G., Alonso-Mori R., Glownia J. M., Nelson S., Sikorski M., Sokaras D., Canton S. E., Lemke H. T., Gaffney K. J. (2019). Finding
Intersections between Electronic Excited State Potential Energy Surfaces
with Simultaneous Ultrafast X-Ray Scattering and Spectroscopy. Chem. Sci..

[39] Sousa C., Domingo A., de Graaf C. (2018). Effect of
Second-Order Spin–Orbit
Coupling on the Interaction between Spin States in Spin-Crossover
Systems. Chem. - Eur. J..

[40] Chábera P., Kjaer K. S., Prakash O., Honarfar A., Liu Y., Fredin L. A., Harlang T. C. B., Lidin S., Uhlig J., Sundström V., Lomoth R., Persson P., Wärnmark K. (2018). FeII Hexa
N-Heterocyclic Carbene Complex with a 528 ps Metal-to-Ligand Charge-Transfer
Excited-State Lifetime. J. Phys. Chem. Lett..

[41] Braun J. D., Lozada I. B., Kolodziej C., Burda C., Newman K. M. E., van Lierop J., Davis R. L., Herbert D. E. (2019). Iron (II) Coordination
Complexes with Panchromatic Absorption and Nanosecond Charge-Transfer
Excited State Lifetimes. Nat. Chem..

[42] Liu L., Duchanois T., Etienne T., Monari A., Beley M., Assfeld X., Haacke S., Gros P. C. (2016). A New Record Excited
State 3MLCT Lifetime for Metalorganic Iron­(II) Complexes. Phys. Chem. Chem. Phys..

[43] Lindh L., Gordivska O., Persson S., Michaels H., Fan H., Chábera P., Rosemann N. W., Gupta A. K., Benesperi I., Uhlig J., Prakash O., Sheibani E., Kjaer K. S., Boschloo G., Yartsev A., Freitag M., Lomoth R., Persson P., Wärnmark K. (2021). Dye-Sensitized Solar Cells Based
on Fe N-Heterocyclic Carbene Photosensitizers with Improved Rod-like
Push-Pull Functionality. Chem. Sci..

[44] Francés-Monerris A., Gros P. C., Assfeld X., Monari A., Pastore M. (2019). Toward Luminescent
Iron Complexes: Unravelling the Photophysics by Computing Potential
Energy Surfaces. ChemPhotoChem.

[45] Lindh L., Chábera P., Rosemann N. W., Uhlig J., Wärnmark K., Yartsev A., Sundström V., Persson P. (2020). Photophysics and Photochemistry
of Iron Carbene Complexes for Solar Energy Conversion and Photocatalysis. Catalysts.

[46] Duchanois T., Liu L., Pastore M., Monari A., Cebrián C., Trolez Y., Darari M., Magra K., Francés-Monerris A., Domenichini E., Beley M., Assfeld X., Haacke S., Gros P. (2018). NHC-Based Iron Sensitizers for DSSCs. Inorganics.

[47] Leis W., Argüello Cordero M. A., Lochbrunner S., Schubert H., Berkefeld A. (2022). A Photoreactive
Iron­(II) Complex
Luminophore. J. Am. Chem. Soc..

[48] Pastore M., Caramori S., Gros P. C. (2024). Iron-Sensitized Solar Cells (FeSSCs). Acc. Chem. Res..

[49] Huber-Gedert M., Nowakowski M., Kertmen A., Burkhardt L., Lindner N., Schoch R., Herbst-Irmer R., Neuba A., Schmitz L., Choi T.-K., Kubicki J., Gawelda W., Bauer M. (2021). Fundamental Characterization,
Photophysics
and Photocatalysis of a Base Metal Iron­(II)-Cobalt­(III) Dyad. Chem. - Eur. J..

[50] Magra K., Domenichini E., Francés-Monerris A., Cebrián C., Beley M., Darari M., Pastore M., Monari A., Assfeld X., Haacke S., Gros P. C. (2019). Impact of the Fac/Mer
Isomerism on the Excited-State Dynamics of Pyridyl-Carbene Fe­(II)
Complexes. Inorg. Chem..

[51] Carrillo U., Viel R., Jamil S. S., Molton F., Duboc C., Perez-Lustres J. L., Heyne K., Haacke S., Cebrian C., Gros P. C. (2025). Fe­(II)
Bidentate Complexes with Long-Lived Triplet
States. Inorg. Chem. Front..

[52] Witas K., Nair S. S., Maisuradze T., Zedler L., Schmidt H., Garcia-Porta P., Rein A. S. J., Bolter T., Rau S., Kupfer S., Dietzek-Ivanšić B., Sorsche D. U. (2024). Beyond the First
Coordination Sphere—Manipulating
the Excited-State Landscape in Iron­(II) Chromophores with Protons. J. Am. Chem. Soc..

[53] Ortiz R. J., Mondal R., McCusker J. K., Herbert D. E. (2025). Leveraging Intramolecular
π-Stacking to Access an Exceptionally Long-Lived 3MC Excited
State in an Fe­(II) Carbene Complex. J. Am. Chem.
Soc..

[54] Riener K., Haslinger S., Raba A., Högerl M. P., Cokoja M., Herrmann W. A., Kühn F. E. (2014). Chemistry
of Iron N-Heterocyclic Carbene Complexes: Syntheses, Structures, Reactivities,
and Catalytic Applications. Chem. Rev..

[55] Liu Y., Kjær K. S., Fredin L. A., Chábera P., Harlang T., Canton S. E., Lidin S., Zhang J., Lomoth R., Bergquist K., Persson P., Wärnmark K., Sundström V. (2015). A Heteroleptic
Ferrous Complex with Mesoionic Bis­(1,2,3-triazol-5-ylidene)
Ligands: Taming the MLCT Excited State of Iron­(II). Chem. - Eur. J..

[56] Carrillo U., Francés-Monerris A., Marri A. R., Cebrián C., Gros P. C. (2022). Substituent-Induced Control of Fac/Mer Isomerism in
Azine-NHC Fe­(II) Complexes. ACS Org. Inorg.
Au.

[57] Francés-Monerris A., Magra K., Darari M., Cebrián C., Beley M., Domenichini E., Haacke S., Pastore M., Assfeld X., Gros P. C., Monari A. (2018). Synthesis and Computational
Study of a Pyridylcarbene Fe­(II) Complex: Unexpected Effects of Fac/Mer
Isomerism in Metal-to-Ligand Triplet Potential Energy Surfaces. Inorg. Chem..

[58] Magra K., Darari M., Domenichini E., Francés-Monerris A., Cebrian C., Beley M., Pastore M., Monari A., Assfeld X., Haacke S., Gros P. C. (2020). Photophysical Investigation
of Iron (II) Complexes Bearing Bidentate Annulated Isomeric Pyridine-NHC
Ligands. J. Phys. Chem. C.

[59] Demirel N., Dawor M., Nadler G., Ivlev S. I., Meggers E. (2024). Stereogenic-at-Iron
Mesoionic Carbene Complex for Enantioselective C–H Amidation. Chem. Sci..

[60] Reuter T., Kruse A., Schoch R., Lochbrunner S., Bauer M., Heinze K. (2021). Higher MLCT Lifetime
of Carbene Iron­(II)
Complexes by Chelate Ring Expansion. Chem. Commun..

[61] Danopoulos A. A., Tsoureas N., Wright J. A., Light M. E. (2004). N-Heterocyclic Pincer
Dicarbene Complexes of Iron­(II): C-2 and C-5 Metalated Carbenes on
the Same Metal Center. Organometallics.

[62] Lincoln, S. F. ; Merbach, A. E. Substitution Reactions of Solvated Metal Ions. In Advances in Inorganic Chemistry; Sykes, A. G. , Ed.; Academic Press: London, 1995; Vol. 42, pp 1–88 10.1016/S0898-8838(08)60051-3.

[63] Merbach A. E. (1987). Use of
High Pressure Kinetic Studies in Determining Inorganic Substitution
Mechanisms. Pure Appl. Chem..

[64] Ducommun Y., Nichols P. J., Merbach A. E. (1989). Variable-Pressure
Kinetic Study of
Formation and Dissociation of Octahedral Divalent Metal Ion Monocomplexes
in N,N-Dimethylformamide: Dissociative Reaction Mechanisms through
the First Row from Manganese to Nickel. Inorg.
Chem..

[65] Saraireh S. A., Altarawneh M. (2014). Thermodynamic Stability and Structures of Iron Chloride
Surfaces: A First-Principles Investigation. J. Chem. Phys..

[66] Sitze M. S., Schreiter E. R., Patterson E. V., Freeman R. G. (2001). Ionic Liquids Based
on FeCl_3_ and FeCl_2_. Raman Scattering and Ab
Initio Calculations. Inorg. Chem..

[67] Inada Y., Hayashi H., Sugimoto K., Funahashi S. (1999). Solvation
Structures of Manganese­(II), Iron­(II), Cobalt­(II), Nickel­(II), Copper­(II),
Zinc­(II), and Gallium­(III) Ions in Methanol, Ethanol, Dimethyl Sulfoxide,
and Trimethyl Phosphate As Studied by EXAFS and Electronic Spectroscopies. J. Phys. Chem. A.

[68] Martínez-Lillo J., Julve M., Brechin E. K. (2015). Hexakis­(Diethylacetamide)­Iron­(II)
Hexahalorhenate­(IV) Ionic Salts: X-Ray Structures and Magnetic Properties. Polyhedron.

[69] Twigg, M. V. ; Burgess, J. 5.4 - Iron. In Comprehensive Coordination Chemistry II; McCleverty, J. A. ; Meyer, T. J. , Eds.; Pergamon: Oxford, 2003; pp 403–553 10.1016/B0-08-043748-6/04208-0.

[70] Cotton S. A. (2018). Iron­(III)
Chloride and Its Coordination Chemistry. J.
Coord. Chem..

[71] Luin U., Arčon I., Valant M. (2022). Structure and Population
of Complex
Ionic Species in FeCl_2_ Aqueous Solution by X-Ray Absorption
Spectroscopy. Molecules.

[72] Baudisch, O. ; Hartung, W. H. ; Milligan, W. O. Tetrapyridino-Ferrous Chloride (Yellow Salt). In Inorganic Syntheses; John Wiley & Sons, 1939; pp 184–185 10.1002/9780470132326.ch64.

[73] Machkour A., Thallaj N. K., Benhamou L., Lachkar M., Mandon D. (2006). The Coordination
Chemistry of FeCl_3_ and FeCl_2_ to Bis­[2-(2,3-Dihydroxyphenyl)-6-Pyridylmethyl]­(2-Pyridylmethyl)­Amine:
Access to a Diiron­(III) Compound with an Unusual Pentagonal-Bipyramidal/Square-Pyramidal
Environment. Chem. - Eur. J..

[74] Housecroft, C. E. ; Sharpe, A. G. Inorganic Chemistry, 5th ed.; Pearson, 2018.

[75] Magra, K. Synthèse et Caractérisation de Nouveaux Complexes Photo-Actifs à Base de Fer, Ph.D. Thesis; Université de Lorraine, 2019 https://hal.univ-lorraine.fr/tel-02877340. (accessed 2025–12–04).

[76] Coe B. J., Glenwright S. J. (2000). Trans-Effects
in Octahedral Transition Metal Complexes. Coord.
Chem. Rev..

[77] McKenzie E. D. (1971). The Steric
Effect in Bis­(2,2′-Bipyridyl) and Bis­(1,10-Phenanthroline)
Metal Compounds. Coord. Chem. Rev..

[78] Goswami S., Mukherjee R., Chakravorty A. (1983). Chemistry of Ruthenium. 12. Reactions
of Bidentate Ligands with Diaquabis [2-(Arylazo) Pyridine] Ruthenium­(II)
Cation. Stereoretentive Synthesis of Tris Chelates and Their Characterization:
Metal Oxidation, Ligand Reduction, and Spectroelectrochemical Correlation. Inorg. Chem..

[79] Goswami S., Chakravarty A. R., Chakravorty A. (1983). Chemistry of Ruthenium. 7. Aqua Complexes
of Isomeric Bis [(2-Arylazo) Pyridine] Ruthenium­(II) Moieties and
Their Reactions: Solvolysis, Protic Equilibriums, and Electrochemistry. Inorg. Chem..

[80] Poli R. (1996). Open-Shell
Organometallics as a Bridge between Werner-Type and Low-Valent Organometallic
Complexes. The Effect of the Spin State on the Stability, Reactivity,
and Structure. Chem. Rev..

[81] Hong Y., Jarrige L., Harms K., Meggers E. (2019). Chiral-at-Iron Catalyst:
Expanding the Chemical Space for Asymmetric Earth-Abundant Metal Catalysis. J. Am. Chem. Soc..

[82] Neese F. (2025). Software Update:
The ORCA Program System—Version 6.0. WIREs Comput. Mol. Sci..

[83] Shiota Y., Sato D., Juhász G., Yoshizawa K. (2010). Theoretical
Study of Thermal Spin Transition between the Singlet State and the
Quintet State in the [Fe­(2-Picolylamine)_3_]^2+^ Spin Crossover System. J. Phys. Chem. A.

[84] Frisch, M. J. ; Trucks, G. W. ; Schlegel, H. B. ; Scuseria, G. E. ; Robb, M. A. ; Cheeseman, J. R. ; Scalmani, G. ; Barone, V. ; Petersson, G. A. ; Nakatsuji, H. ; Li, X. ; Caricato, M. ; Marenich, A. V. ; Bloino, J. ; Janesko, B. G. ; Gomperts, R. ; Mennucci, B. ; Hratchian, H. P. ; Ortiz, J. V. ; Izmaylov, A. F. ; Sonnenberg, J. L. ; Williams-Young, D. ; Ding, F. ; Lipparini, F. ; Egidi, F. ; Goings, J. ; Peng, B. ; Petrone, A. ; Henderson, T. ; Ranasinghe, D. ; Zakrzewski, V. G. ; Gao, J. ; Rega, N. ; Zheng, G. ; Liang, W. ; Hada, M. ; Ehara, M. ; Toyota, K. ; Fukuda, R. ; Hasegawa, J. ; Ishida, M. ; Nakajima, T. ; Honda, Y. ; Kitao, O. ; Nakai, H. ; Vreven, T. ; Throssell, K. ; Montgomery, J. A., Jr. ; Peralta, J. E. ; Ogliaro, F. ; Bearpark, M. J. ; Heyd, J. J. ; Brothers, E. N. ; Kudin, K. N. ; Staroverov, V. N. ; Keith, T. A. ; Kobayashi, R. ; Normand, J. ; Raghavachari, K. ; Rendell, A. P. ; Burant, J. C. ; Iyengar, S. S. ; Tomasi, J. ; Cossi, M. ; Millam, J. M. ; Klene, M. ; Adamo, C. ; Cammi, R. ; Ochterski, J. W. ; Martin, R. L. ; Morokuma, K. ; Farkas, O. ; Foresman, J. B. ; Fox, D. J. Gaussian 16; Gaussian, Inc.: Wallingford CT, 2016.

[85] Darari M., Domenichini E., Francés-Monerris A., Cebrián C., Magra K., Beley M., Pastore M., Monari A., Assfeld X., Haacke S., Gros P. C. (2019). Iron­(II) Complexes
with Diazinyl-NHC Ligands: Impact of π-Deficiency of the Azine
Core on Photophysical Properties. Dalton Trans..

[86] Darari M., Francés-Monerris A., Marekha B., Doudouh A., Wenger E., Monari A., Haacke S., Gros P. C. (2020). Towards
Iron­(II) Complexes with Octahedral Geometry: Synthesis, Structure
and Photophysical Properties. Molecules.

[87] Francés-Monerris A., Gros P. C., Pastore M., Assfeld X., Monari A. (2019). Photophysical
Properties of Bichromophoric Fe­(II) Complexes Bearing an Aromatic
Electron Acceptor. Theor. Chem. Acc..

[88] Isayev O., Gorb L., Leszczynski J. (2007). Theoretical
Calculations: Can Gibbs
Free Energy for Intermolecular Complexes Be Predicted Efficiently
and Accurately?. J. Comput. Chem..

[89] Mennucci B. (2012). Polarizable
Continuum Model. WIREs Comput. Mol. Sci..

[90] Grimme S., Antony J., Ehrlich S., Krieg H. (2010). A Consistent and Accurate
Ab Initio Parametrization of Density Functional Dispersion Correction
(DFT-D) for the 94 Elements H-Pu. J. Chem. Phys..

[91] Reiher M., Salomon O., Artur Hess B. (2001). Reparameterization
of Hybrid Functionals
Based on Energy Differences of States of Different Multiplicity. Theor. Chem. Acc..

[92] Diez-Cabanes V., Prampolini G., Francés-Monerris A., Monari A., Pastore M. (2020). Iron’s
Wake: The Performance of Quantum Mechanical-Derived
Versus General-Purpose Force Fields Tested on a Luminescent Iron Complex. Molecules.

[93] Lee C., Yang W., Parr R. G. (1988). Development of the Colle-Salvetti
Correlation-Energy Formula into a Functional of the Electron Density. Phys. Rev. B.

[94] Perdew J. P. (1986). Density-Functional
Approximation for the Correlation Energy of the Inhomogeneous Electron
Gas. Phys. Rev. B.

[95] Stephens P. J., Devlin F. J., Chabalowski C. F., Frisch M. J. (1994). Ab Initio Calculation
of Vibrational Absorption and Circular Dichroism Spectra Using Density
Functional Force Fields. J. Phys. Chem. A.

[96] Adamo C., Barone V. (1999). Toward Reliable Density Functional Methods without
Adjustable Parameters: The PBE0Model. J. Chem.
Phys..

[97] Zhao Y., Truhlar D. G. (2008). The M06 Suite of Density Functionals for Main Group
Thermochemistry, Thermochemical Kinetics, Noncovalent Interactions,
Excited States, and Transition Elements: Two New Functionals and Systematic
Testing of Four M06-Class Functionals and 12 Other Function. Theor. Chem. Acc..

[98] Chai J.-D., Head-Gordon M. (2008). Long-Range
Corrected Hybrid Density Functionals with
Damped Atom–Atom Dispersion Corrections. Phys. Chem. Chem. Phys..

[99] Yu H. S., He X., Li S. L., Truhlar D. G. (2016). MN15: A Kohn–Sham Global-Hybrid
Exchange–Correlation Density Functional with Broad Accuracy
for Multi-Reference and Single-Reference Systems and Noncovalent Interactions. Chem. Sci..

[100] Riplinger C., Neese F. (2013). An Efficient and near
Linear Scaling
Pair Natural Orbital Based Local Coupled Cluster Method. J. Chem. Phys..

[101] Klamt A., Schüürmann G. (1993). COSMO: A New Approach
to Dielectric Screening in Solvents with Explicit Expressions for
the Screening Energy and Its Gradient. J. Chem.
Soc., Perkin Trans. 2.

[102] Barone V., Cossi M. (1998). Quantum Calculation of Molecular
Energies and Energy Gradients in Solution by a Conductor Solvent Model. J. Phys. Chem. A.

[103] Pápai M., Vankó G., de Graaf C., Rozgonyi T. (2013). Theoretical
Investigation of the Electronic Structure of Fe­(II) Complexes at Spin-State
Transitions. J. Chem. Theory Comput..

[104] Zhu X., Thompson K. C., Martínez T. J. (2019). Geodesic
Interpolation for Reaction
Pathways. J. Chem. Phys..

