# Supplementary material for: Synthetic Mechanism of a Fe(II) N‑Heterocyclic Carbene Bidentate Complex Revealed by Electronic Structure Methods
Source: Inorg Chem. 2026 Jun 22;65(26):15136–49. doi: 10.1021/acs.inorgchem.6c02095 (PMC13343516; doi:10.1021/acs.inorgchem.6c02095)
Supplement: Supplementary file 1 [file ic6c02095_si_001.pdf]

Supporting Information for:

Synthetic Mechanism of a Fe(II) *N*-Heterocyclic Carbene Bidentate Complex Revealed by Electronic Structure Methods

Abdelazim M. A. Abdelgawwad,[a] Ulises Carrillo[b,], Philippe C. Gros[b], Cristina Cebrián[c] and Antonio Francés-Monerris[a,*]

a) Institut de Ciència Molecular, Universitat de València, P.O. Box 22085, València 46071, Spain

**A.F.-M.: antonio.frances@uv.es*

b) Université de Caen Normandie, CERMN UR 4258, 14000 Caen, France

c) Université de Grenoble Alpes, CNRS, DCM, F-38000 Grenoble

d) Université de Strasbourg, CNRS, ICS, F-67000 Strasbourg, France

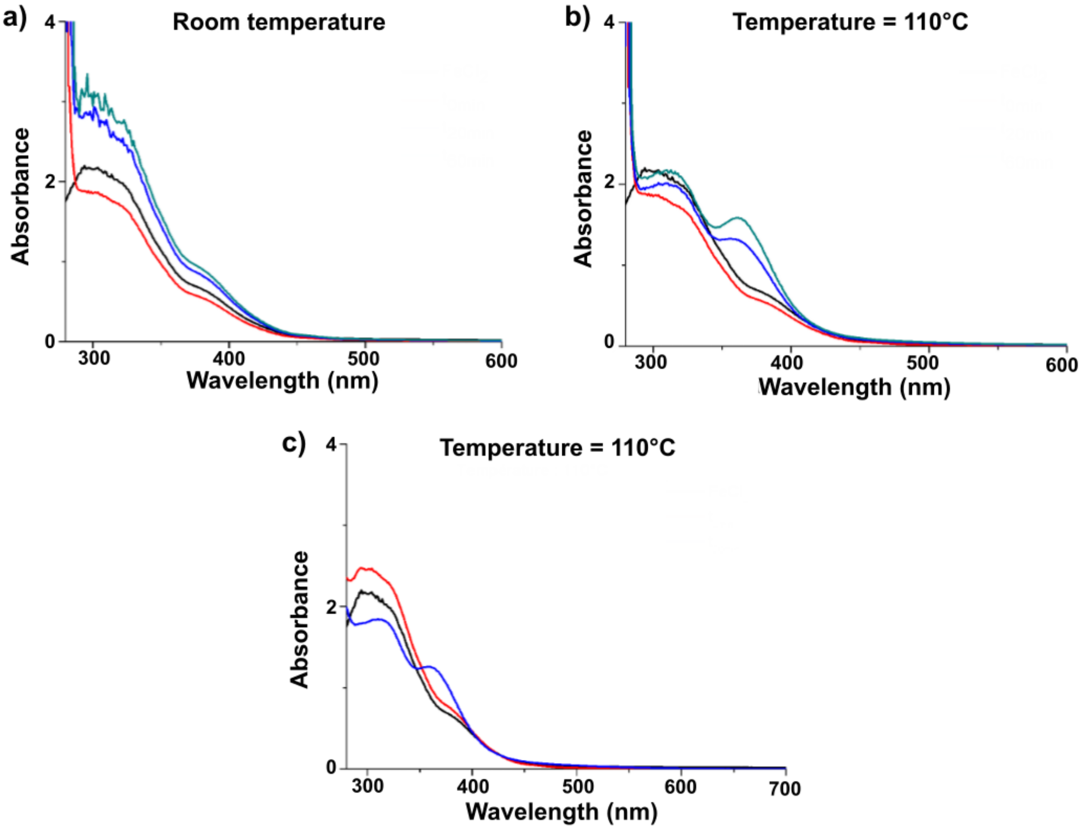

Figure S1: Qualitative UV-visible spectroscopy monitoring at 0 min (red), 20 min (blue) and 60 min (green) of the pre-coordination of **HL0** to $FeCl_2$ in DMF (a) at room temperature and (b) at 110 °C. The UV-vis spectrum of iron chloride in DMF (black) is also shown for comparison. In panel (c), the experience conducted in (b) was repeated with an equimolar concentration of pyridine instead of **HL0** at room temperature. Retrieved and translated from the PhD thesis by Kévin Magra, "Synthèse et caractérisation de nouveaux complexes photo-actifs à base de fer", Université de Lorraine (France), 2019. Available at https://hal.univ-lorraine.fr/tel-02877340 (accessed December 6th, 2025).

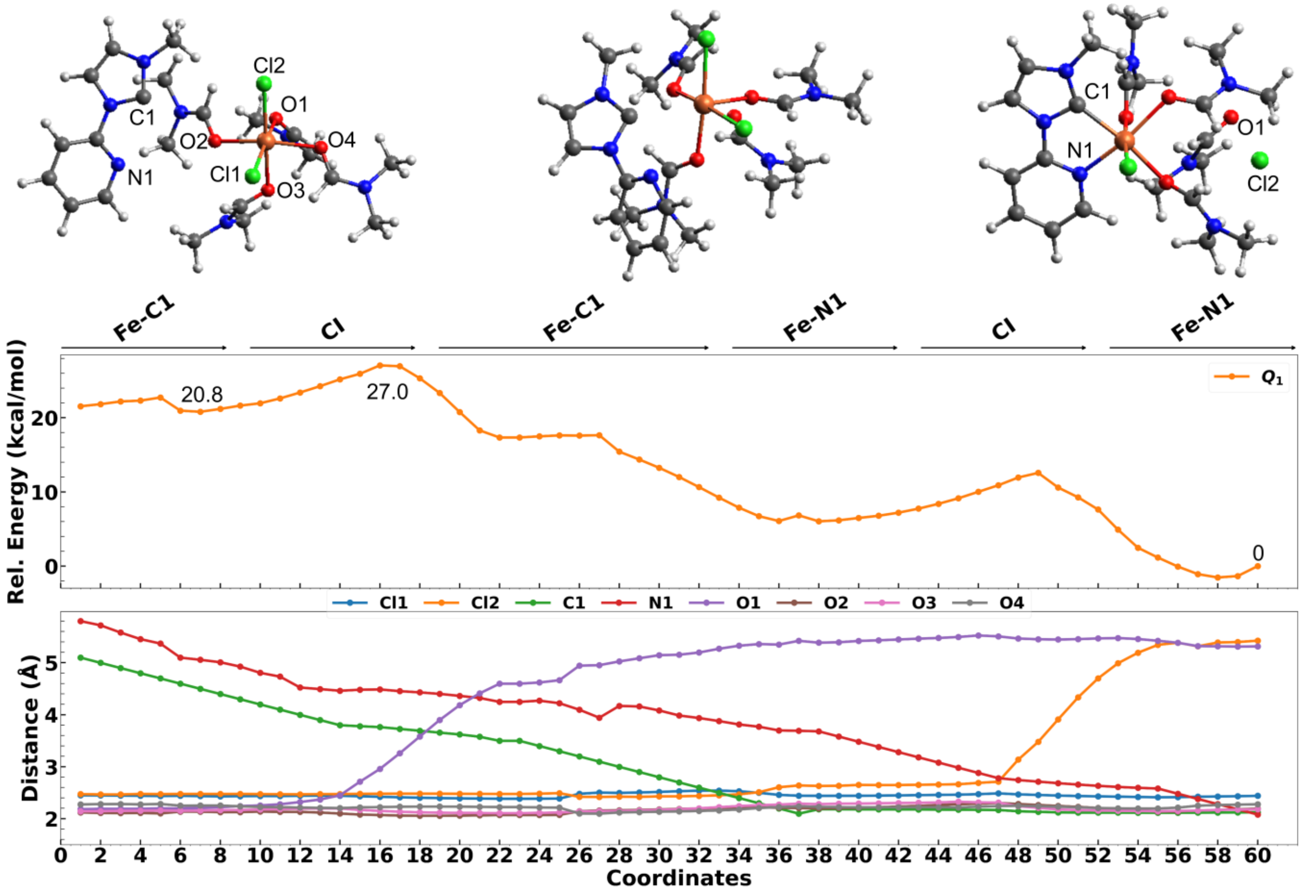

Figure S2. B3LYP/6-31+G(d,p) energy profile for the *cis*-$[FeCl_2(DMF)_4]$ + C^N → $[FeCl(C^\wedge N)(DMF)_3]^+$ + DMF + Cl^- reaction in the Q_1 state. Fe-C1 indicates a relaxed scan optimization of the Fe-C1 distance and Fe-N1 for the Fe-N1 distance. CI refers to coordinate interpolation. Bond distances are defined between the atom defined in the label and the Fe center.

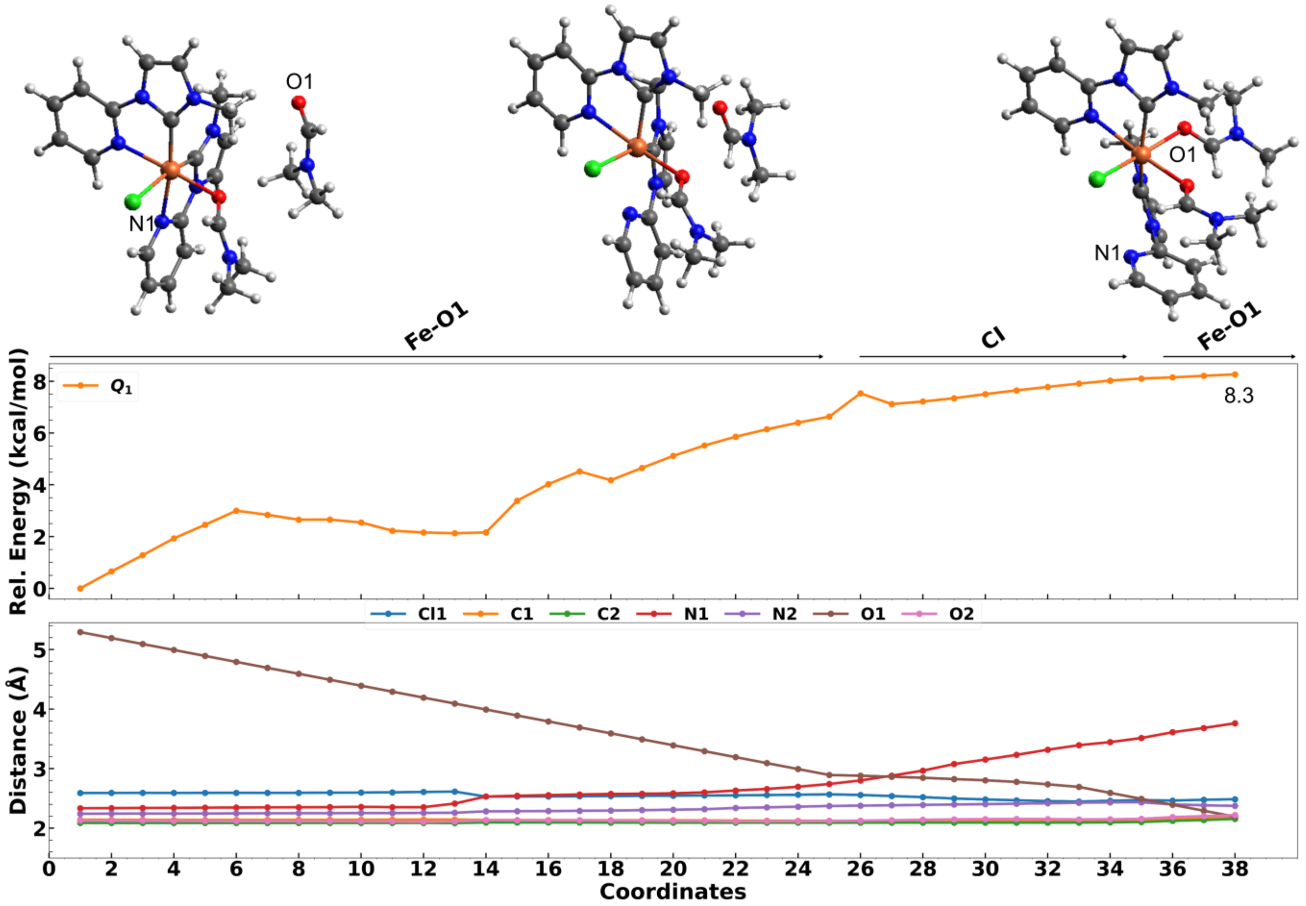

Figure S3. B3LYP/6-31+G(d,p) energy profile for the $[FeCl(C^{\wedge}N)_2DMF]^+$ + DMF → *cis,xxx*-$[Fe(C^{\wedge}N)_2(DMF)_2]^{2+}$ + Cl^- reaction in the quintet state (Q_1). Fe-O1 indicates a relaxed scan optimization of the Fe-O1 distance and CI refers to coordinate interpolation. Bond distances are defined between the atom defined in the label and the Fe center. The Cl^- group is never released.

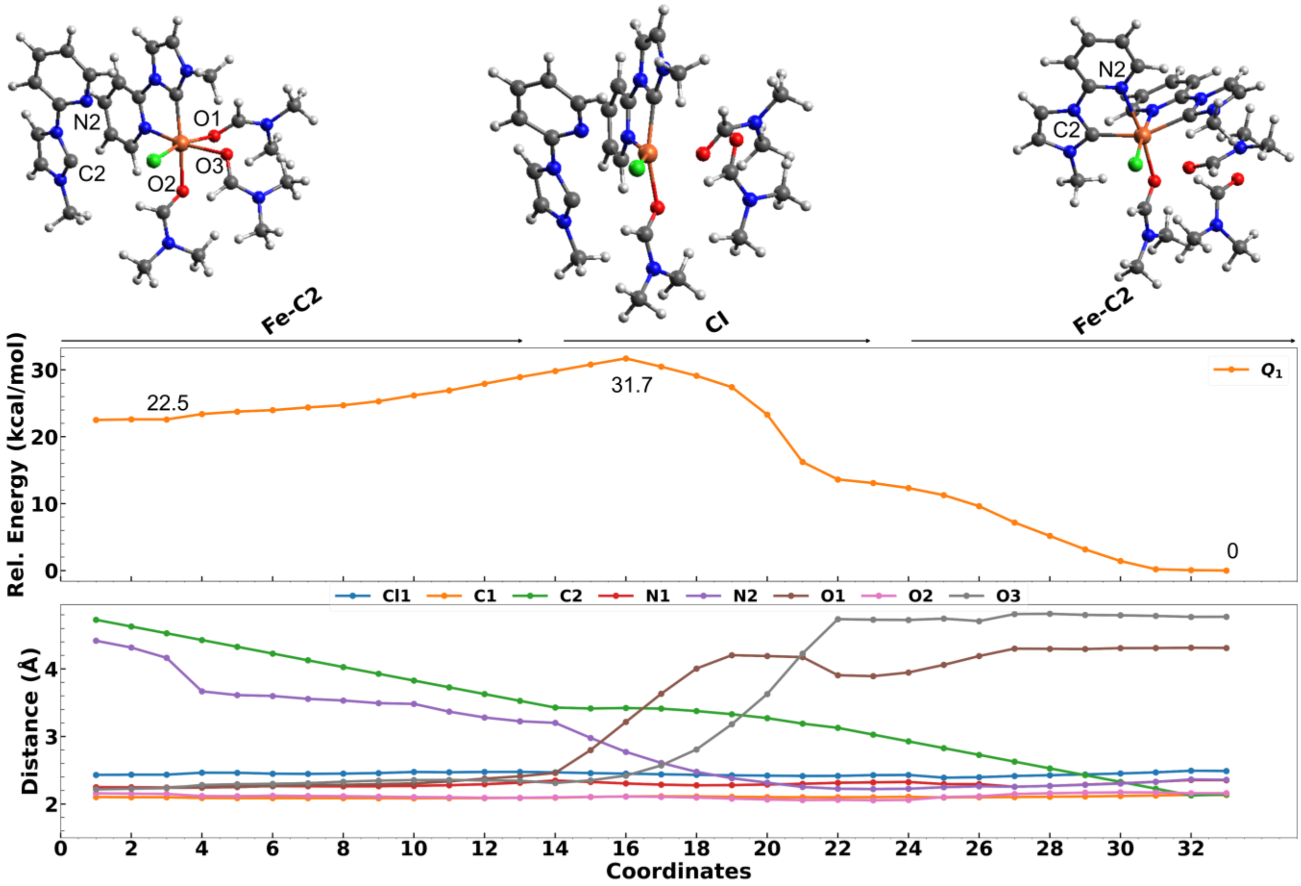

Figure S4. B3LYP/6-31+G(d,p) energy profile for the $[FeCl(C\string^N)(DMF)_3]^+$ + C^N → $[FeCl(C\string^N)_2DMF]^+$ (**B1**) + 2 DMF reaction in the Q_1 state. Fe-C2 indicates a relaxed scan optimization of the Fe-C2 distance. CI refers to coordinate interpolation. Bond distances are defined between the atom defined in the label and the Fe center.

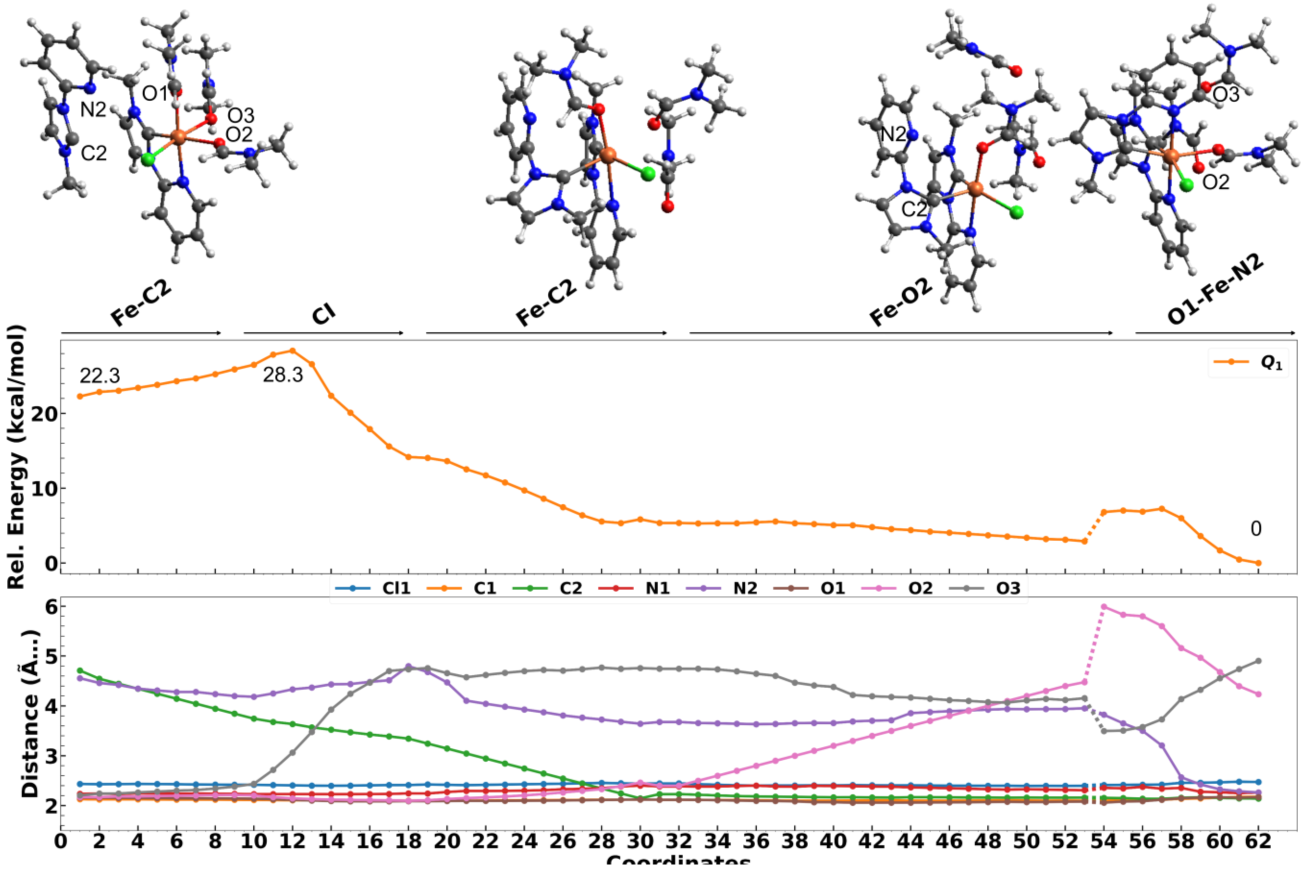

Figure S5. B3LYP/6-31+G(d,p) energy profile for the $[FeCl(C^\wedge N)(DMF)_3]^+$ + C^N → $[FeCl(C^\wedge N)_2DMF]^+$ (**C1**) + 2 DMF reaction in the Q_1 state. Fe-C2 indicates a relaxed scan optimization of the Fe-C2 distance, Fe-N2 for the Fe-N2 distance, and O1-Fe-N2 for the O1-Fe-N2 angle. CI refers to coordinate interpolation. Bond distances are defined between the atom defined in the label and the Fe center.

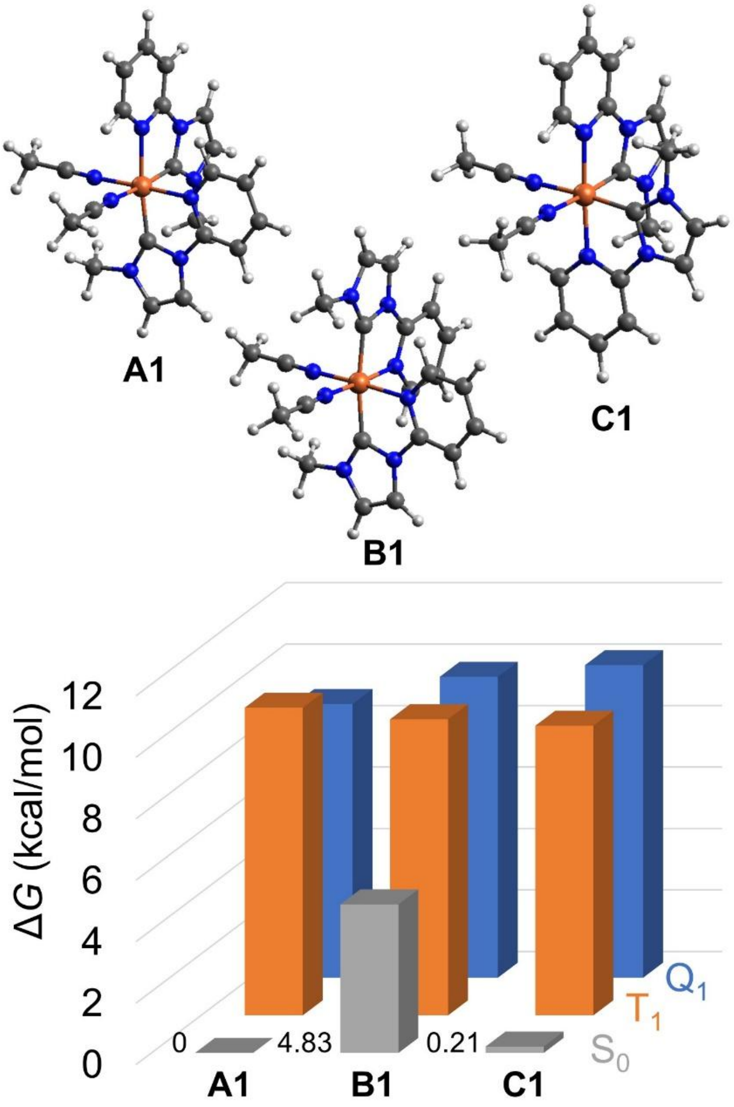

Figure S6. Δ*G* values for the $[Fe(CH_3CN)_2(C^\wedge N)_2]^{2+}$ compounds reported by Witas *et al*, *J. Am. Chem. Soc.* **2024**, *146* (29), 19710–19719. Gibbs energies are computed with the B3LYP/6-31G* method and all values are relative to the most stable isomer (**A1**) in the S_0 state.

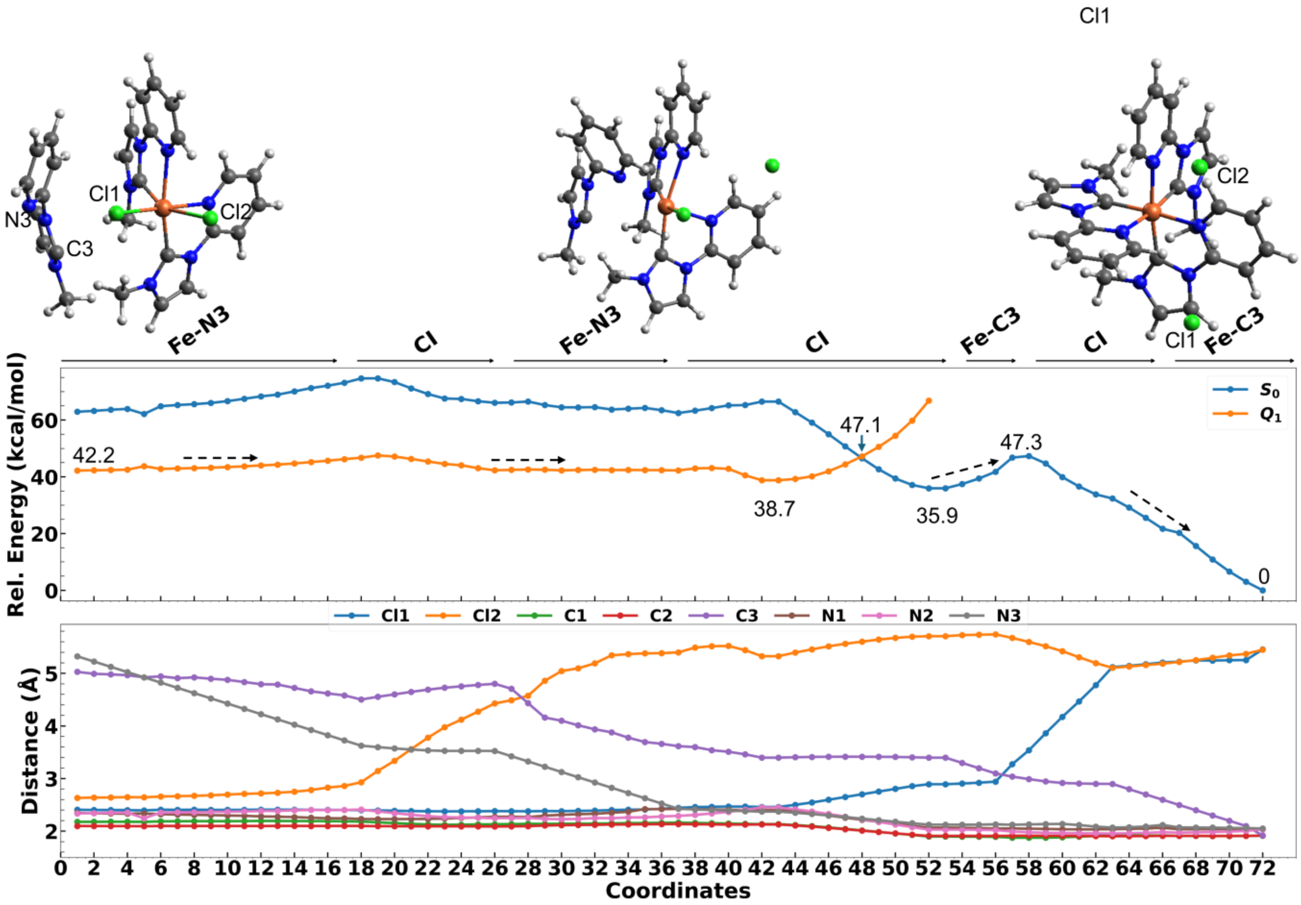

Figure S7. B3LYP/6-31+G(d,p) complete energy profile for the FAC-ID-2 path yielding *fac*-**C0** through and I_d mechanism. The reaction corresponds to *cis,xxx*-[$FeCl_2$(C^N)$_2$] + C^N → *fac*-[Fe(C^N)$_3$]$^{2+}$ + 2 Cl^-. Fe-N3 indicates a relaxed scan optimization of the Fe-N3 distance and Fe-C3 for the Fe-C2 distance. CI refers to coordinate interpolation. The bottom panel plots the distances between the Fe center and the indicated atom throughout the path. The dotted arrows indicate the reaction path.

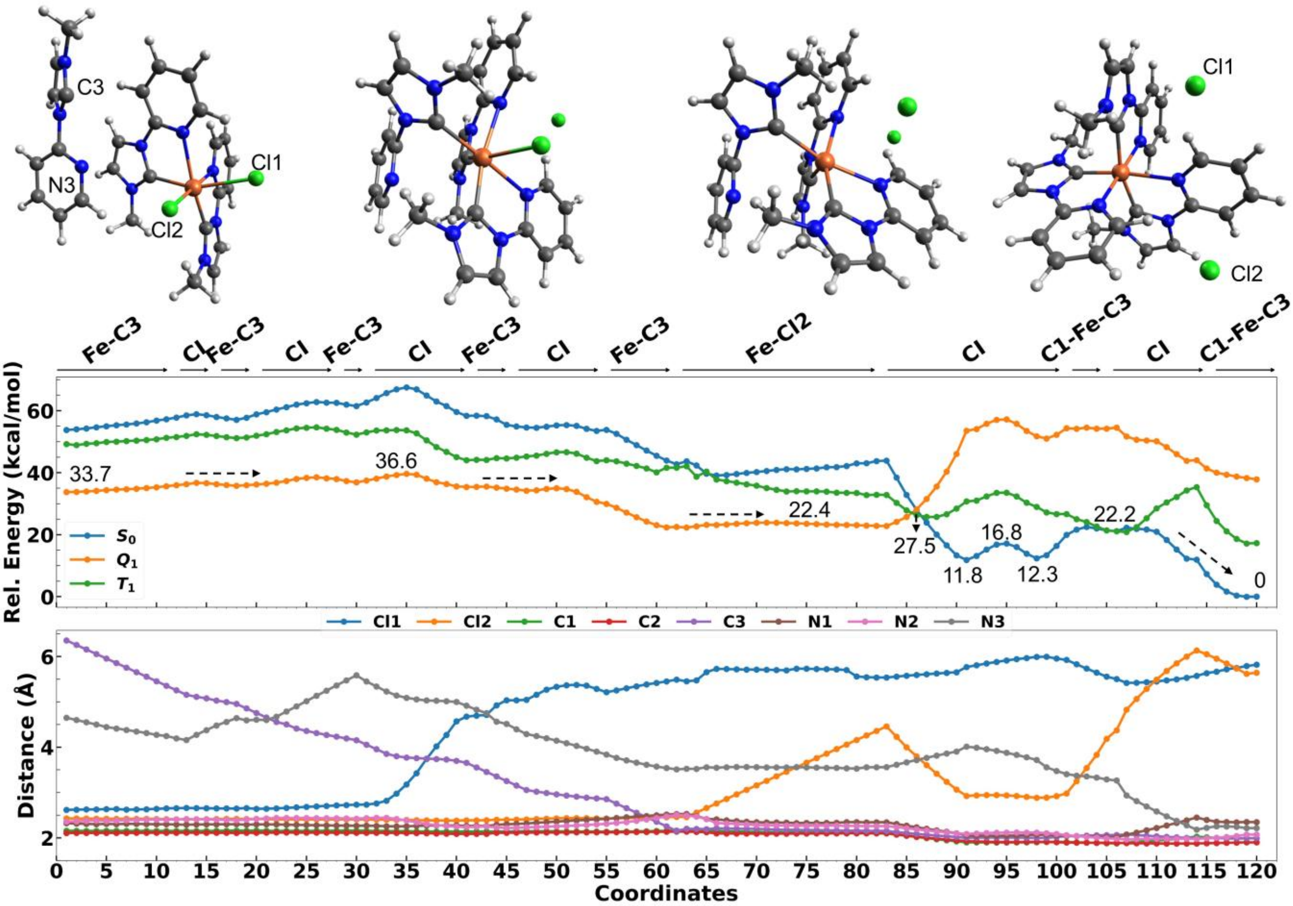

Figure S8. B3LYP/6-31+G(d,p) complete energy profile for the MER-ID-1 path yielding *mer*-**C0** through and I_d mechanism. The reaction corresponds to *cis,xxx*-$[FeCl_2(C\text{^}N)_2]$ + C^N → *mer*-$[Fe(C\text{^}N)_3]^{2+}$ + 2 Cl^-. Fe-N3 indicates a relaxed scan optimization of the Fe-N3 distance and Fe-C3 for the Fe-C2 distance. CI refers to coordinate interpolation. The bottom panel plots the distances between the Fe center and the indicated atom throughout the path. The dotted arrows indicate the reaction path.

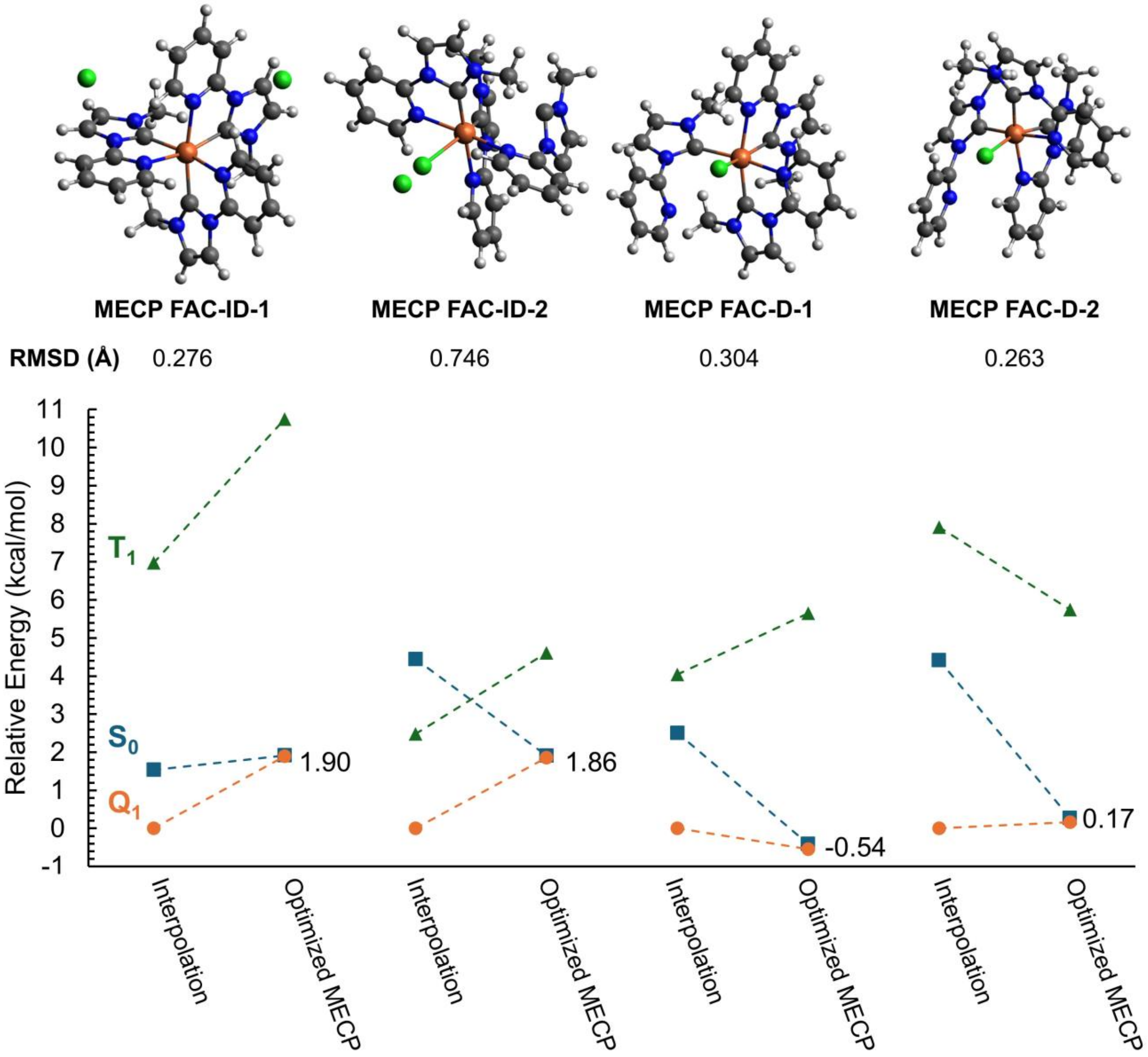

Figure S9. Minimum energy crossing point (MECP) optimization of the Q_1/S_0 crossing estimated through coordinate interpolation. For each path, all energies are relative to the Q_1 state at the interpolation estimate, to facilitate comparisons. All results correspond to the B3LYP/6-31G* level of theory, the method used to optimize the structures. The RMSD between the interpolated structures and the optimized MECPs is also shown.

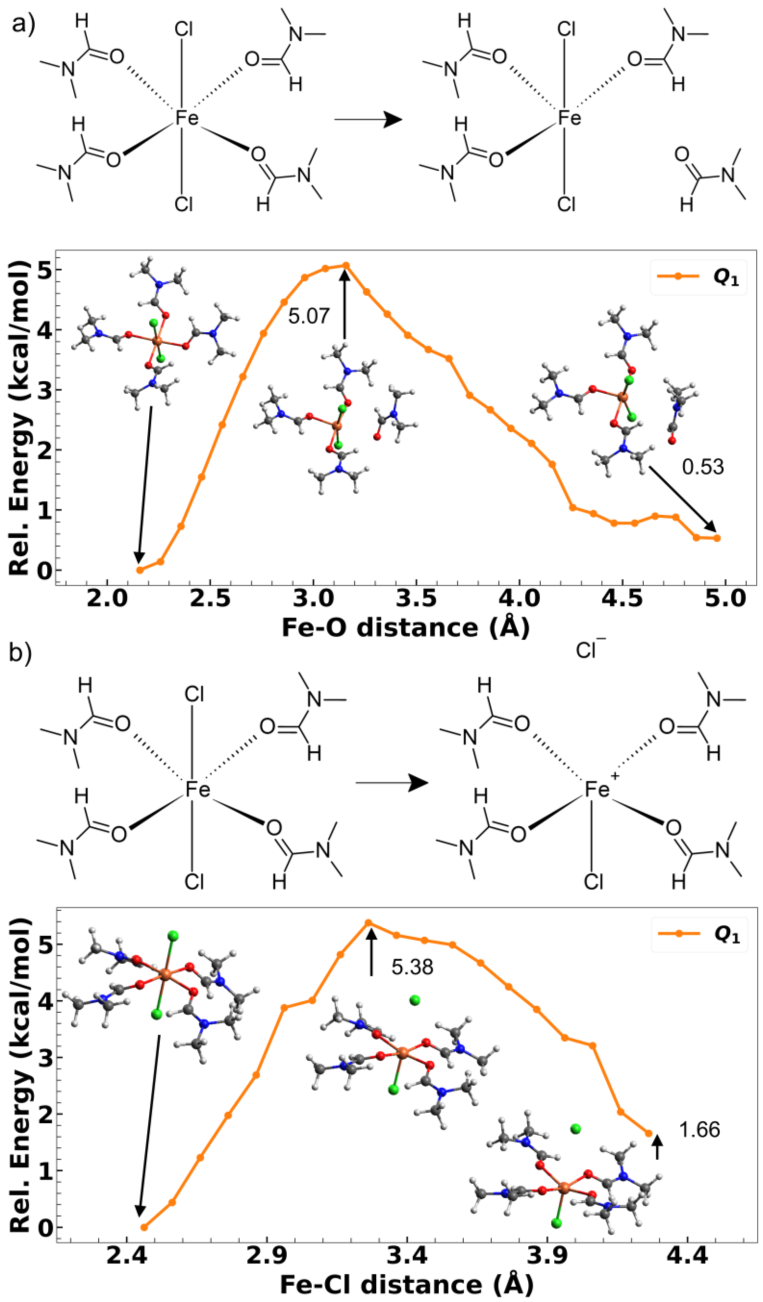

Figure S10. B3LYP/6-31+G(d,p) relaxed scan energy profile for the Fe–O (panel a) and Fe–Cl (panel b) bond break of $[FeCl_2(DMF)_4]$ in the Q_1 state. The profiles correspond to reaction 6a, *trans*-$[FeCl_2(DMF)_4]$ → $[FeCl_2(DMF)_3]$ + DMF (panel a), and reaction 6b, *trans*-$[FeCl_2(DMF)_4]$ → $[FeCl(DMF)_4]^+$ + Cl^- (panel b).

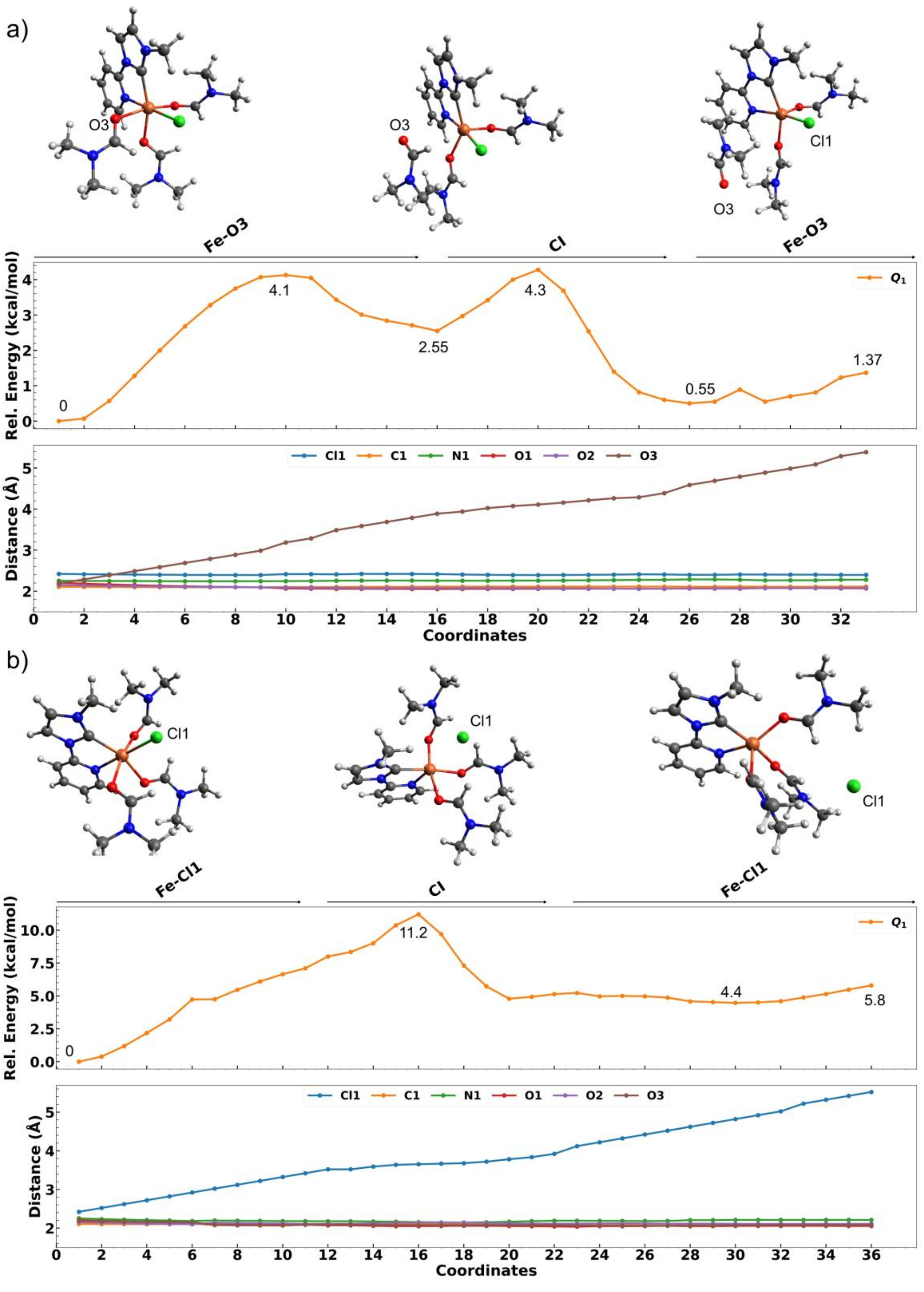

Figure S11. B3LYP/6-31+G(d,p) complete energy profile for reaction 8a (panel a) and 8b (panel b) in the Q_1 state. Fe-O3 and Fe-Cl1 indicate relaxed scan optimizations of the Fe-O3 and Fe-Cl1 distances, respectively. CI refers to coordinate interpolation.

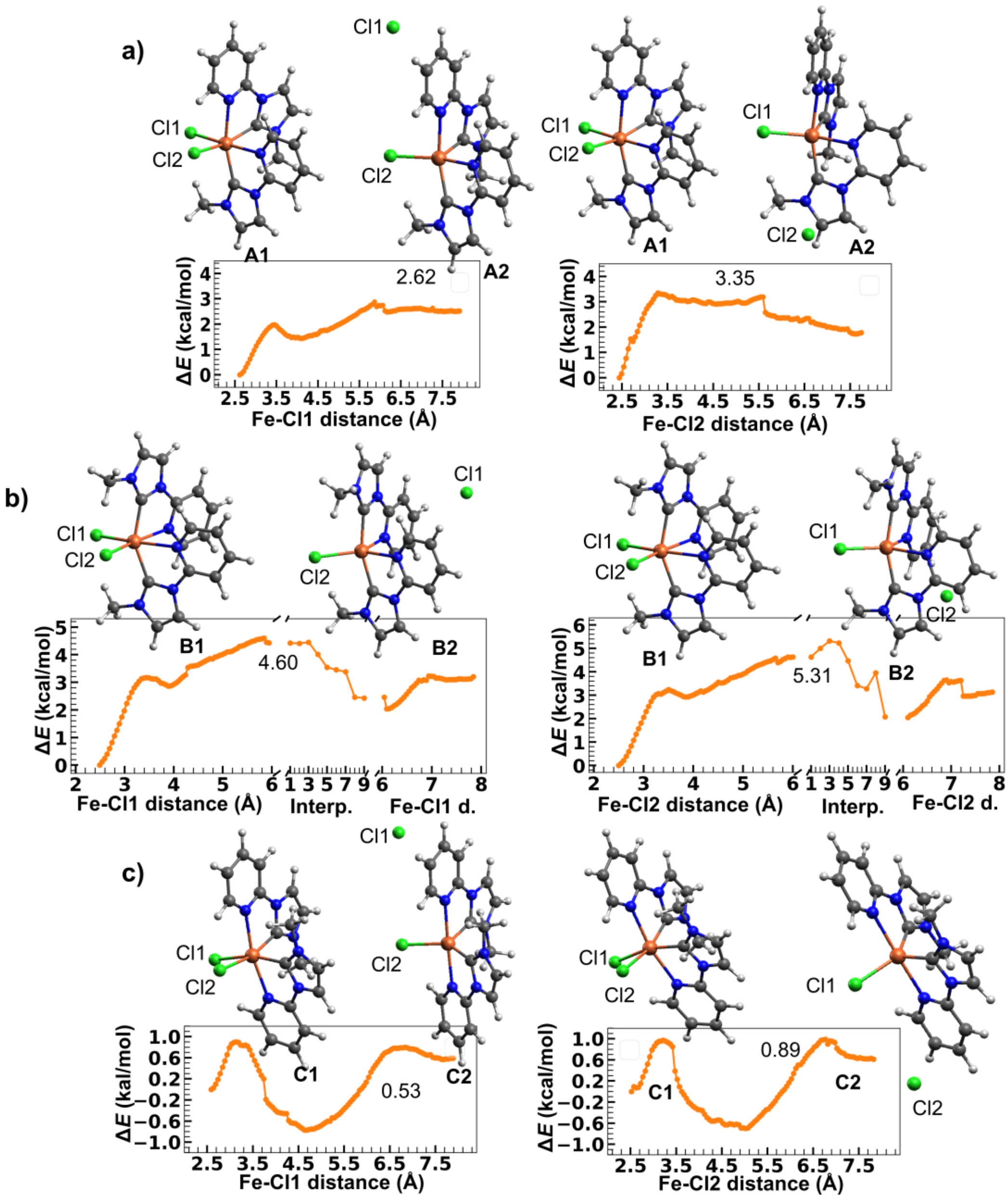

Figure S12. B3LYP/6-31+G(d,p) reaction profiles for the $[FeCl_2(C\text{^}N)_2]$ → $[FeCl(C\text{^}N)_2]^+$ + Cl^- process in the Q_1 state. Panels a), b), and c) correspond to the Cl^- loss reactions of the intermediates **A1**, **B1**, and **C1**, respectively.

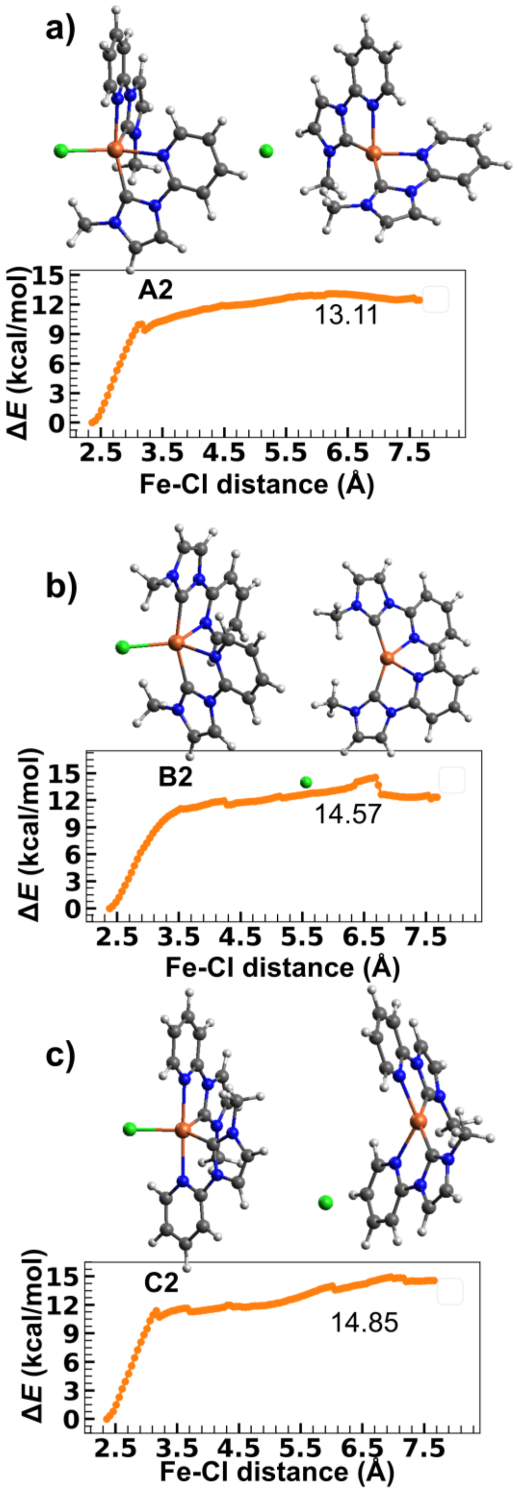

Figure S13. B3LYP/6-31+G(d,p) reaction profiles (relaxed scans) for the $[FeCl(C\text{^}N)_2]^+ \rightarrow [Fe(C\text{^}N)_2]^{2+} + Cl^-$ (reaction 12) in the Q_1 state. Panels a), b), and c) correspond to the Cl^- loss reactions of the intermediates **A2**, **B2**, and **C2**, respectively.

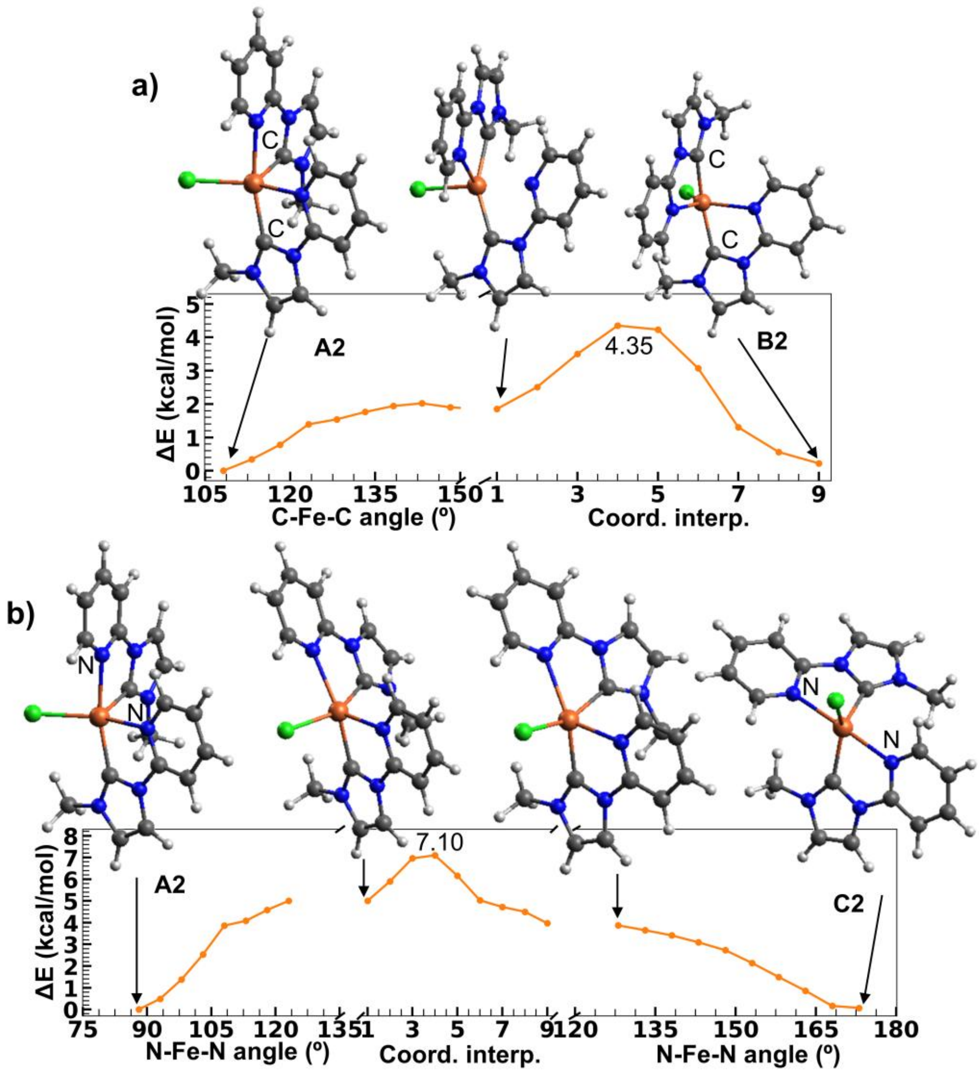

Figure S14. B3LYP/6-31+G(d,p) reaction profiles for a) **A2↔B2** and b) **A2↔C2** isomerizations obtained through relaxed scans of C–Fe–C and N–Fe–N angles and coordinate interpolations in the Q_1 state.

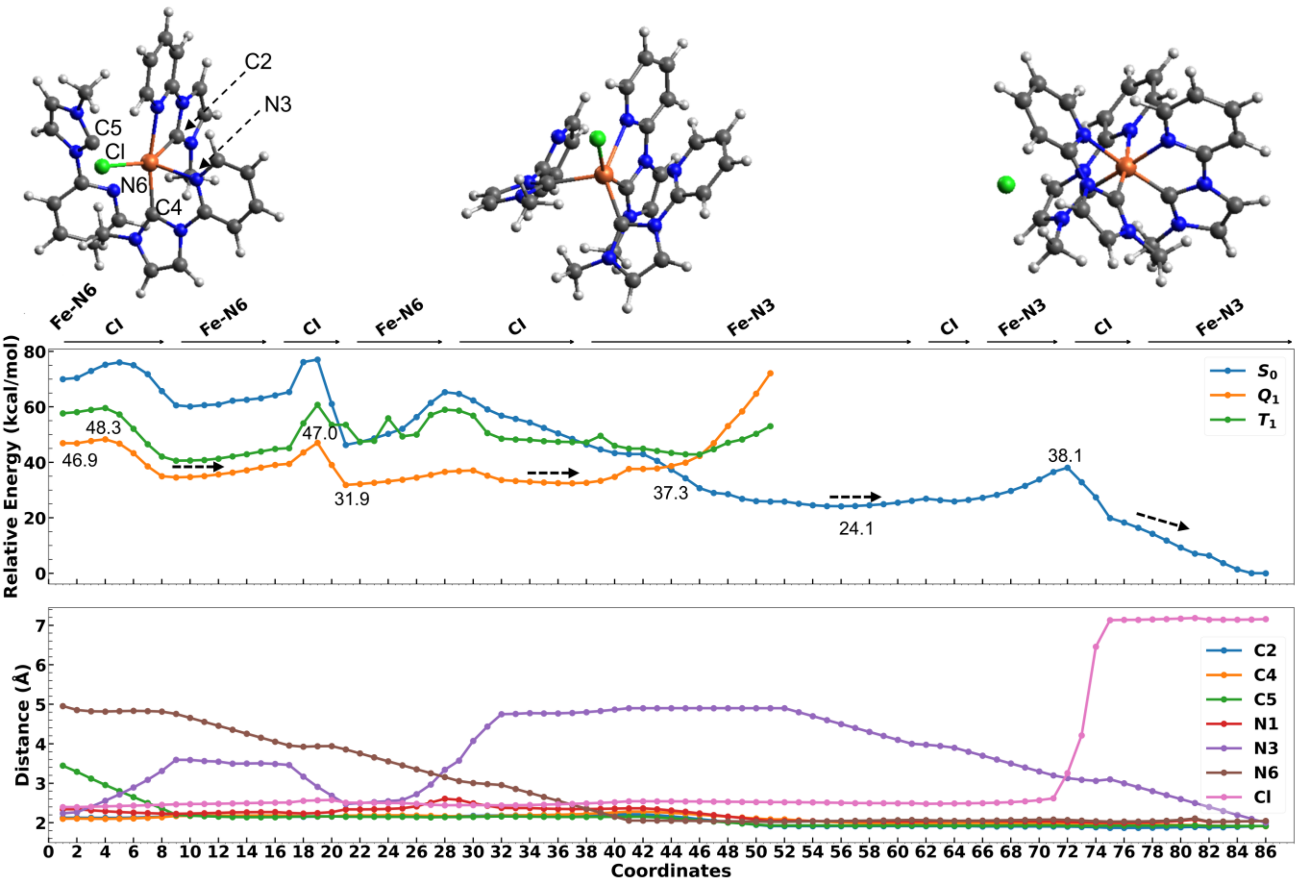

Figure S15. B3LYP/6-31+G(d,p) complete energy profile for the FAC-D-2 path, yielding *fac*-**C0** through the reaction $[FeCl(C^\wedge N)_2]^+$ + C^N → $[Fe(C^\wedge N)_3]^{2+}$ + Cl^-. Profiles obtained through combinations of relaxed scan calculations of Fe–N6 (in the Q_1 state) and Fe–N3 (in the S_0 state) bonds, and coordinate interpolations (CI). The bottom panel plots the distances between the Fe center and the indicated atom throughout the path. The dotted arrows indicate the reaction path.

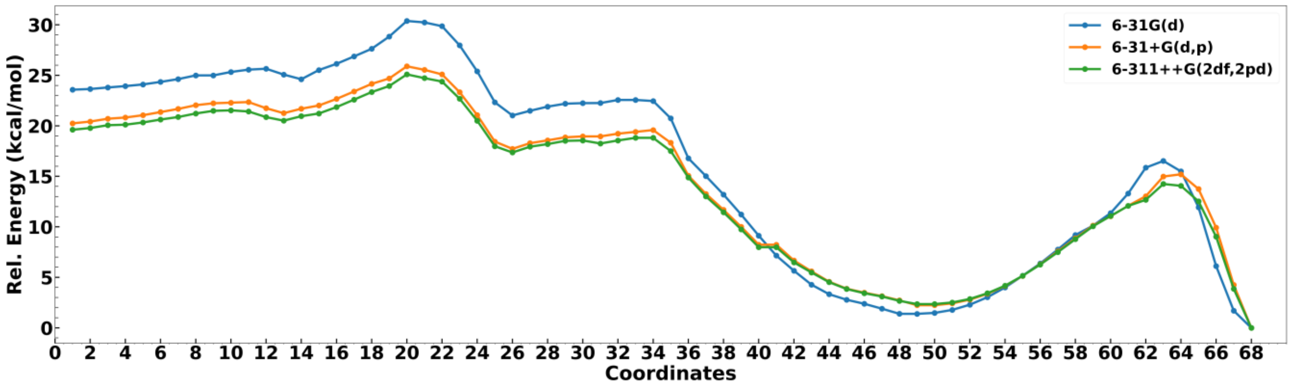

Figure S16. Evaluation of the basis set effect in the *trans*-[$FeCl_2(DMF)_4$] + C^N → *trans*,*cis*-[FeCl(C^N)$(DMF)_3$]$^+$ + DMF + Cl^- transformation (reaction 2, Figure 1 of the main text) in the quintet state. Geometries have been optimized only with the B3LYP/6-31G* method.

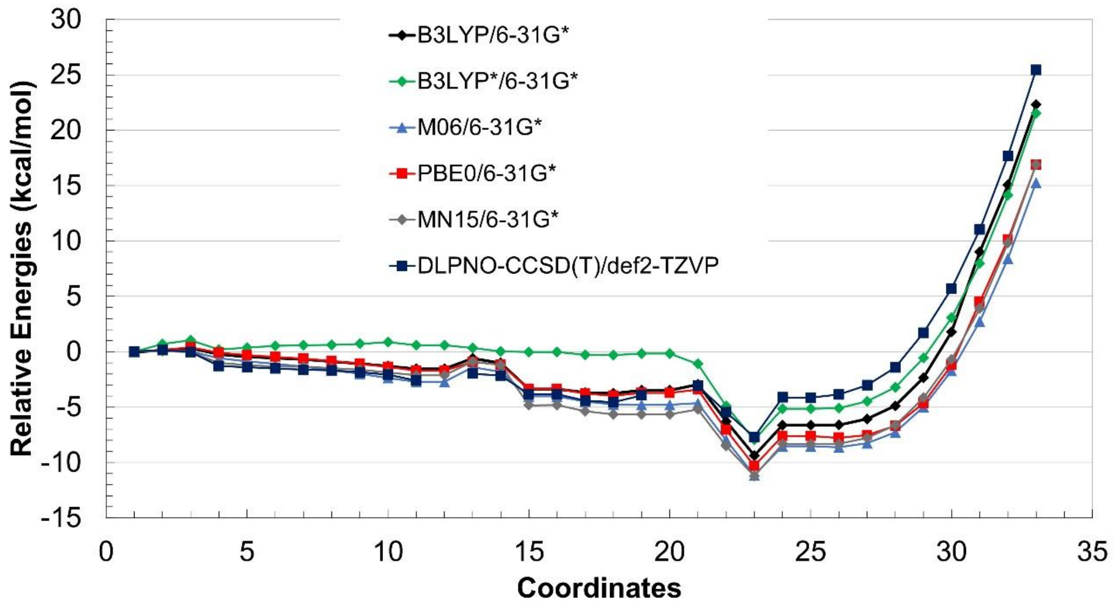

Figure S17. Fragment of the Q_1 energy profile corresponding to path FAC-D-1 (Figure 6 of the main article) computed with different electronic structure methods. Energies of each profile are relative to their corresponding coordinate 1.

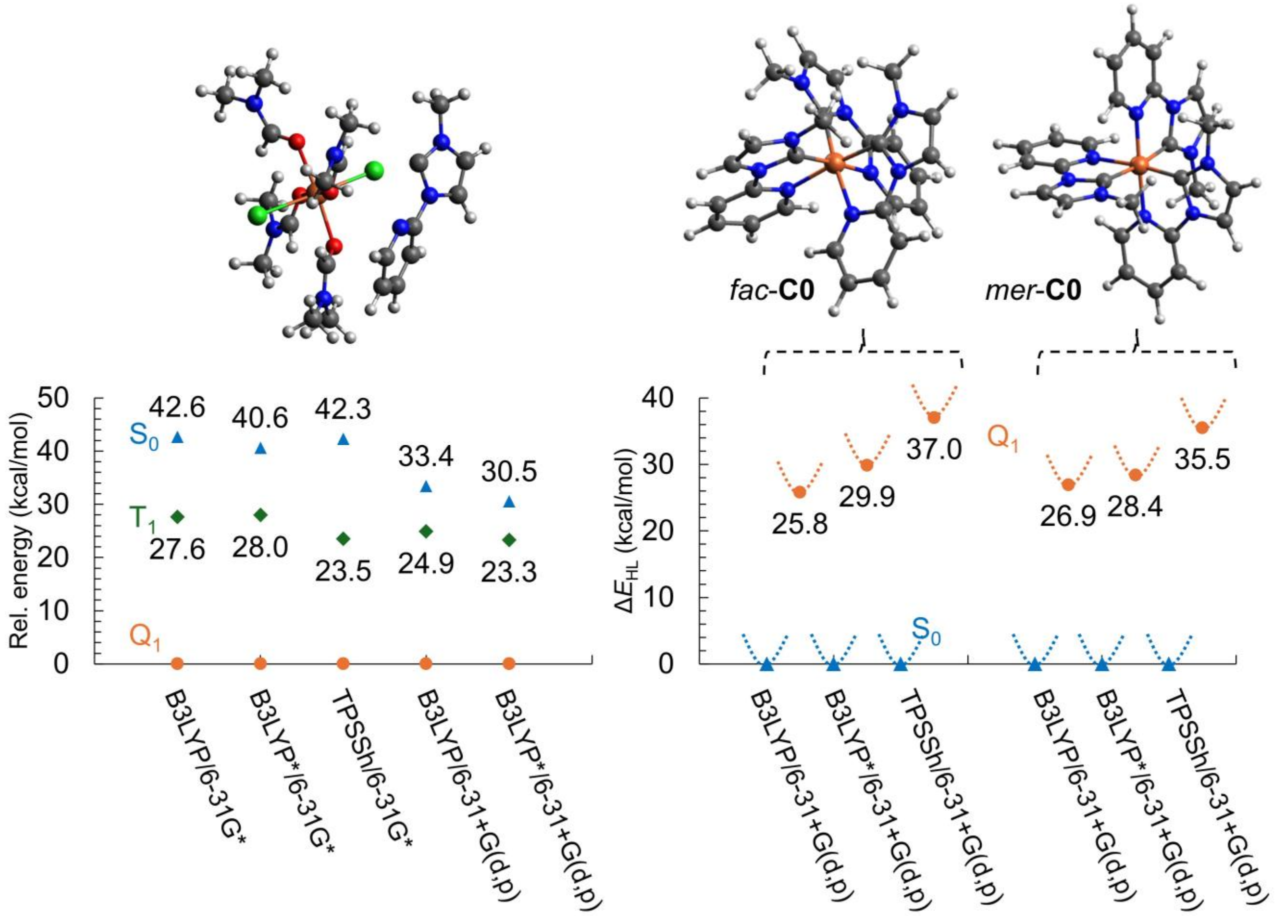

Figure S18. State ordering validation. The left panel shows the S_0, T_1, and Q_1 energies (relative to Q_1) for the reactants of reaction 2 (coordinate 1 of Figure 1, main text), computed vertically. The right panel shows the spin-state transition $\Delta E_{HL} = E_{HS} - E_{LS}$ computed for *fac* and *mer* isomers of **C0**, where each geometry have been optimized with the corresponding functional and the 6-31G* basis set, and final energies are refined with the 6-31+G(d,p) basis set.

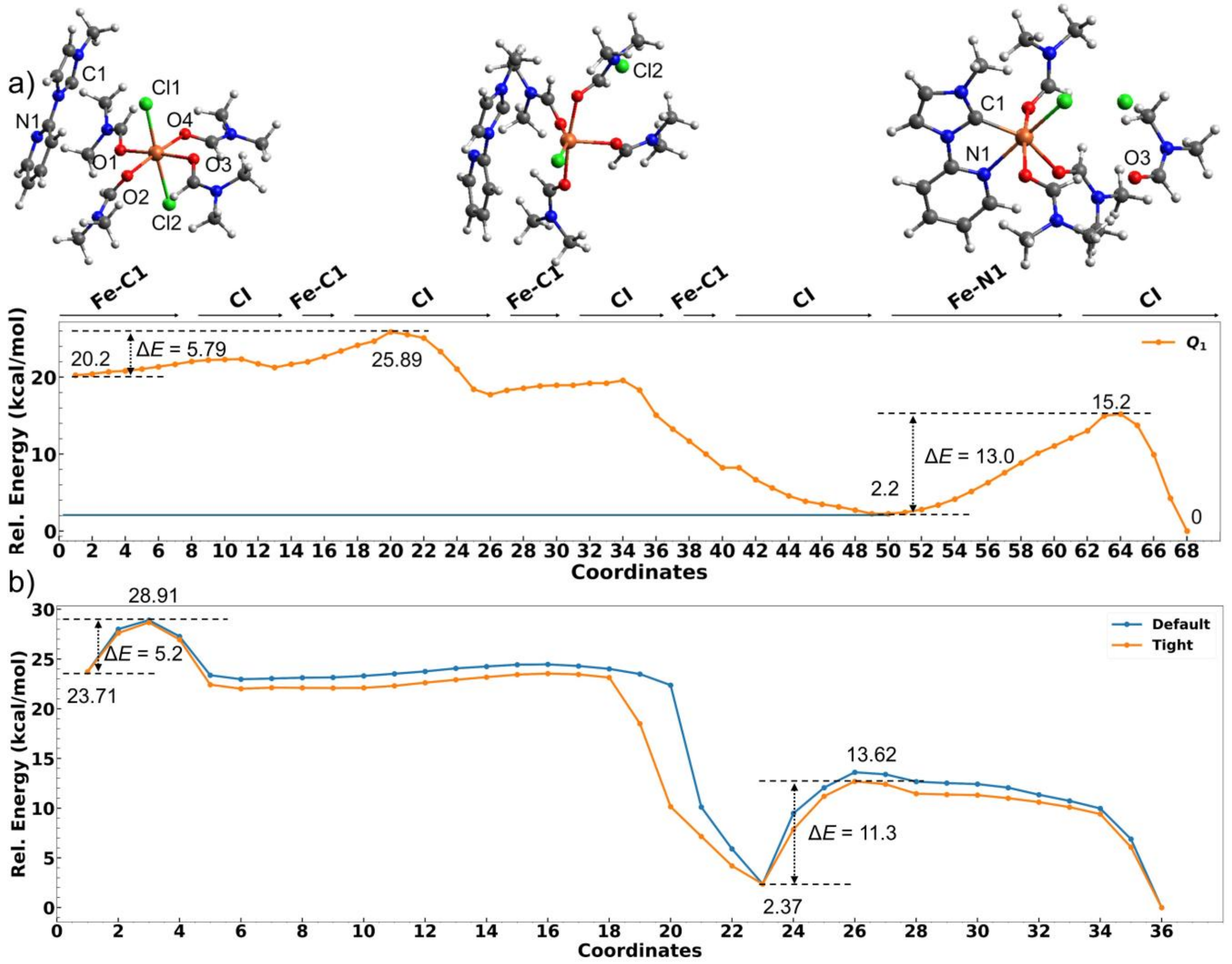

Figure S19. Reaction **2** computed with the relaxed scans + coordinate interpolation method (panel a) and the minimum energy path (MEP) by means of the nudge elastic band (NEB) method (panel b). Two different convergence thresholds, default (Tol_MaxFP_I = 10^{-3} and Tol_RMSFP_I = $5 \cdot 10^{-4}$ Eh/Bohr) and tight (Tol_MaxFP_I = $5 \cdot 10^{-4}$ and Tol_RMSFP_I = $2.5 \cdot 10^{-4}$ Eh/Bohr), have been used. The XYZ coordinates of the MEPs are published as electronic SI.

Table S1. Δ*G* (in kcal/mol, T = 298.15 K, p = 1 atm) at different electronic states for the $FeCl_2$ and *cis*/*trans*-[$FeCl_2(DMF)_4$] molecules in DMF solution. Geometries optimized for each state with the B3LYP/6-31G* method. *cis*/*trans*-[$FeCl_2(DMF)_4$] energies relative to *trans*-[$FeCl_2(DMF)_4$] in the Q_1 state.

**Electronic state**	**$FeCl_2$**	***cis*-[$FeCl_2(DMF)_4$]**	***trans*-[$FeCl_2(DMF)_4$]**
		B3LYP/6-31G*	
Q_1	0	1.49	0
T_1	39.81	27.98	23.66
S_0	79.42	38.14	37.57
		DLPNO-CCSD(T)/def2-TZVP[a]	
Q_1	0	0.97	0
T_1	–[b]		
S_0	89.35		

[a]Contribution to the Gibbs energy computed with the B3LYP/6-31G* method.
[b]Not converged.

Table S2. Δ*G* (in kcal/mol) for [$Fe(NHC)_2(Cl)_2$] and [$Fe(NHC)_2(Cl)$]$^+$ intermediates (T = 298.15 K, p = 1 atm).

	**S_0**	**T_1**	**Q_1**
B3LYP/6-31G*			
**A1**	8.01	7.09	0
**B1**	13.39	6.03	0.09
**C1**	4.78	8.68	2.63
**A2**	3.08	10.76	0.02
**B2**	16.51	3.33	0.06
**C2**	8.04	9.18	0
DLPNO-CCSD(T)/def2-VTZP[a]			
**A1**	16.92	21.44	0.16
**B1**	22.40	18.24	0
**C1**	13.39	21.04	2.71
**A2**	29.62	29.80	1.75
**B2**	32.76	16.77	2.38
**C2**	22.78	22.27	0

[a] Thermal correction to Gibbs free energy computed at the B3LYP/6-31G* level of theory.

Table S3. Energy (*E*), enthalpy (*H*), and Gibbs energy (*G*) differences between products and reactants for the *fac* and *mer* paths pathways computed in this work at 298K. Energies in kcal/mol. Average Δ*G* – Δ*E* value is given with its associated standard deviation.

	**Δ*E***	**Δ*H***	**Δ*G***	**Δ*G* - Δ*E***
**FAC-ID-1**	-43.71	-41.63	-37.76	5.95
**FAC-ID-2**	-41.01	-38.83	-34.38	6.63
**FAC-D-1**	-42.31	-40.33	-34.56	7.75
**FAC-D-2**	-46.71	-44.94	-39.22	7.49
**MER-ID-1**	-33.25	-29.42	-24.75	8.51
**Average**				7.27±1
